# Prevalence, diversity and applications potential of nodules endophytic bacteria: a systematic review

**DOI:** 10.3389/fmicb.2024.1386742

**Published:** 2024-05-15

**Authors:** Mohamed Hnini, Jamal Aurag

**Affiliations:** Microbiology and Molecular Biology Team, Center of Plant and Microbial Biotechnology, Biodiversity and Environment, Faculty of Sciences, Mohammed V University in Rabat, Rabat, Morocco

**Keywords:** non-rhizobial endophytic bacteria, legumes, diversity, PGP properties, coinoculation

## Abstract

Legumes are renowned for their distinctive biological characteristic of forming symbiotic associations with soil bacteria, mostly belonging to the *Rhizobiaceae* familiy, leading to the establishment of symbiotic root nodules. Within these nodules, rhizobia play a pivotal role in converting atmospheric nitrogen into a plant-assimilable form. However, it has been discerned that root nodules of legumes are not exclusively inhabited by rhizobia; non-rhizobial endophytic bacteria also reside within them, yet their functions remain incompletely elucidated. This comprehensive review synthesizes available data, revealing that *Bacillus* and *Pseudomonas* are the most prevalent genera of nodule endophytic bacteria, succeeded by *Paenibacillus*, *Enterobacter*, *Pantoea*, *Agrobacterium*, and *Microbacterium*. To date, the bibliographic data available show that *Glycine max* followed by *Vigna radiata, Phaseolus vulgaris* and *Lens culinaris* are the main hosts for nodule endophytic bacteria. Clustering analysis consistently supports the prevalence of *Bacillus* and *Pseudomonas* as the most abundant nodule endophytic bacteria, alongside *Paenibacillus*, *Agrobacterium*, and *Enterobacter*. Although non-rhizobial populations within nodules do not induce nodule formation, their presence is associated with various plant growth-promoting properties (PGPs). These properties are known to mediate important mechanisms such as phytostimulation, biofertilization, biocontrol, and stress tolerance, emphasizing the multifaceted roles of nodule endophytes. Importantly, interactions between non-rhizobia and rhizobia within nodules may exert influence on their leguminous host plants. This is particularly shown by co-inoculation of legumes with both types of bacteria, in which synergistic effects on plant growth, yield, and nodulation are often measured. Moreover these effects are pronounced under both stress and non-stress conditions, surpassing the impact of single inoculations with rhizobia alone.

## 1 Introduction

By 2050, the global population is anticipated to surpass 9 billion, experiencing significant growth from the current 7 billion (Kumar and Dubey, 2020). This escalating population, coupled with climate change-induced abiotic and biotic pressures and land scarcity, is adversely affecting worldwide food production. In response, the primary objective of crop nutrient management is to enhance productivity, addressing the escalating demand for food associated with population growth (Yeboah et al., 2021). However, the intensified use of chemical fertilizers and pesticides, driven by population-mediated agricultural pressure, has deleterious environmental consequences (Adhikary et al., 2022). These include disruptions in soil physicochemical properties, texture, porosity, water-holding capacity, soil acidification, water eutrophication, and disturbance of beneficial soil microbial flora (Baweja et al., 2020). Additionally, chemical inputs have direct implications for human health, leading to issues such as cancers and nutritional deficiencies (Schindler and Hecky, 2009). Remarkably, despite the application of fertilizers, a substantial proportion, exceeding 50%, is lost to the environment, with organo-mineral fertilizers having the highest environmental impact, with water consumption, fossil resource use, and global warming potential (Litskas, 2023).

In response to these challenges, urgent measures are imperative to secure yields while prioritizing environmental sustainability. Among the most promising agro-ecological alternatives are biofertilization, biological pest control, and the cultivation of legumes. Beyond their traditional roles, legumes provide ecological services such as enhancing agro-system resilience and sustainability (Powers and Thavarajah, 2019). Notably, legumes exhibit a remarkable ability to establish a symbiotic association with soil bacteria, termed rhizobia (Legume Nodulating Bacteria or LNB), resulting in the formation of root nodules. Within these nodules, atmospheric nitrogen is converted into ammonia, a usable nitrogen form for the plant (Stagnari et al., 2017). This process offers significant agroecological benefits by reducing the need for synthetic nitrogen fertilizers and other inputs responsible for soil and environmental degradation. Furthermore, the legumes nitrogen-fixing symbiosis contributes to soil fertility, serving as a source of protein-rich foods. Legumes, therefore, play a vital role in addressing the global demand for protein, enhancing soil fertility, and thriving in challenging environmental conditions.

Given these considerations, a high number of studies have focused on legumes and their interaction with LNB to better understand their advantages and leverage the benefits of symbiosis (Rodríguez-Rodríguez et al., 2021). These studies explore a diverse range of legume species, revealing substantial diversity in the associated rhizobia. The effectiveness of nodulation relies on the efficiency and competitiveness of the rhizobia strains (Andrews and Andrews, 2017). However, under stressful environmental conditions, competition between rhizobia strains and the rhizospheric bacterial population can result in ineffective nodulation (Mwenda et al., 2023). In this context, Legumes, like other plants, interact with a diverse microbiome, including plant growth-promoting rhizobacteria (PGPR), to mitigate environmental stress impacts. PGPR play a crucial role in enhancing plant growth and health, offering benefits beyond stress mitigation.

PGPR in general, have been advocated as an effective solution to address the environmental repercussions of chemical intensive agriculture. PGPR can enhance plant growth through various mechanisms, including improved nutrient availability, growth stimulation, enhanced stress tolerance, and protection against phytopathogens. While most of these bacteria are typical rhizospheric inhabitants, some, termed endophytes, exhibit the capability to penetrate root epidermis, colonize root tissues, and migrate to various plant parts and organs (Strobel and Daisy, 2003). Furthermore, endophytic bacteria have been isolated from various plant parts, including roots, xylem vessels, leaves, flowers, and fruits. The term “endophytes” was first coined by De Bary (1866) to refer to microbes, such as bacteria, fungi, cyanobacteria, and actinomycetes, residing within plant tissues (Rana et al., 2020). These endophytes have garnered attention for their beneficial effects on host plants, including improved growth and health. Mechanisms involved in these effects encompass the production of phytohormones or siderophores (Złoch et al., 2016), enhanced metal absorption, inorganic phosphate solubilization, secretion of biologically active compounds, and induction of resistance to phytopathogens and parasites (Spence and Bais, 2015).

In legumes, endophytes have recently been isolated from root nodules induced by rhizobia, prompting inquiries into their identity, diversity, prevalence in different legume species, establishment mechanisms in nodules, and their role within the rhizobium-legume symbiosis. This knowledge gap emphasizes the need for further exploration to unravel the complexities surrounding these endophytes.

Nodule endophytes were termed opportunists, as they are thought to infect nodules when rhizobia induce nodule formation (Leite et al., 2017). Diverse nomenclature, such as bacteria associated with the nodule (Rajendran et al., 2012), non-symbiotic bacteria with disinfected nodules (Costa et al., 2013), nodule endophytes (Velázquez et al., 2013), non-rhizobial endophytes (De Meyer et al., 2015), non-rhizobial bacteria (Dhole et al., 2016), and more recently Non-Nodular Endophytic Bacteria (NNEB) (Ríos-Ruiz et al., 2019). Noteworthy genera of nodules’ non-rhizobial endophytic bacteria in legumes include *Agrobacterium, Klebsiella, Paenibacillus, Bacillus, Blastobacter, Dyadobacter, Phyllobacterium, Pseudomonas, Ensifer,* and *Enterobacter* (De Meyer et al., 2015), *Aerobacter, Aeromonas, Chryseomonas, Curtobacterium, Erwinia, Flavimonas,* and *Sphingomonas* in pea cultivars (Elvira-Recuenco and van Vuurde, 2000), *Arthrobacter, Acinetobacter, Micromonospora, Mycobacterium,* and *Stenotrophomonas* (Velázquez et al., 2013), *Cupriavidus, Providencia, Staphylococcus, Kocuria, Micrococcus, Frondihabitans, Paracoccus* and *Roseomonas* (Leite et al., 2017). Some of these non-rhizobial bacteria have been shown to benefit plants through various activities, such as synthesizing plant hormones, fixing atmospheric N_2_, solubilizing inorganic phosphate, and enhancing the catalytic activity of ACC deaminase, underscoring their potential for sustainable agriculture (Kour et al., 2020). Despite these documented benefits, there is a dearth of information regarding nodule endophytes across various ecological niches, including desert plants. This lack of comprehensive understanding extends to their diversity, activities valorization, and effects on plant tolerance and growth under stressful conditions, presenting a notable gap in current research.

This review addresses the exploration of legume-nodule-associated endophytic bacteria unveils their diversity, ecological roles, and benefits for plants under varied conditions. The review synthesizes data, identifying *Bacillus* and *Pseudomonas* as most prevalent endophytes, primarily hosted by *Glycine max*. Non-rhizobial populations within nodules exhibit plant growth-promoting properties, indicating their potential importance in plant growth and/or resilience, and their significance in sustainable agriculture. The interaction dynamics between rhizobial and non-rhizobial bacteria within nodules, influencing leguminous host plants, add complexity to the symbiotic relationship. Co-inoculation with non-rhizobial endophytes shows synergistic effects on plant growth, surpassing the impact of single rhizobia inoculations. The primary objective of this literature review was to identify patterns and specificities in the relationship between legume species and nodule endophyte bacteria species. Through this comprehensive approach, the study aimed to provide valuable insights into the intricate dynamics of the legume-nodule endophytic bacteria relationship and its contribution in sustainable agriculture, emphasizing the intricate interplay within the legume-nodule ecosystem.

## 2 Research methodology

**Figure 1:**
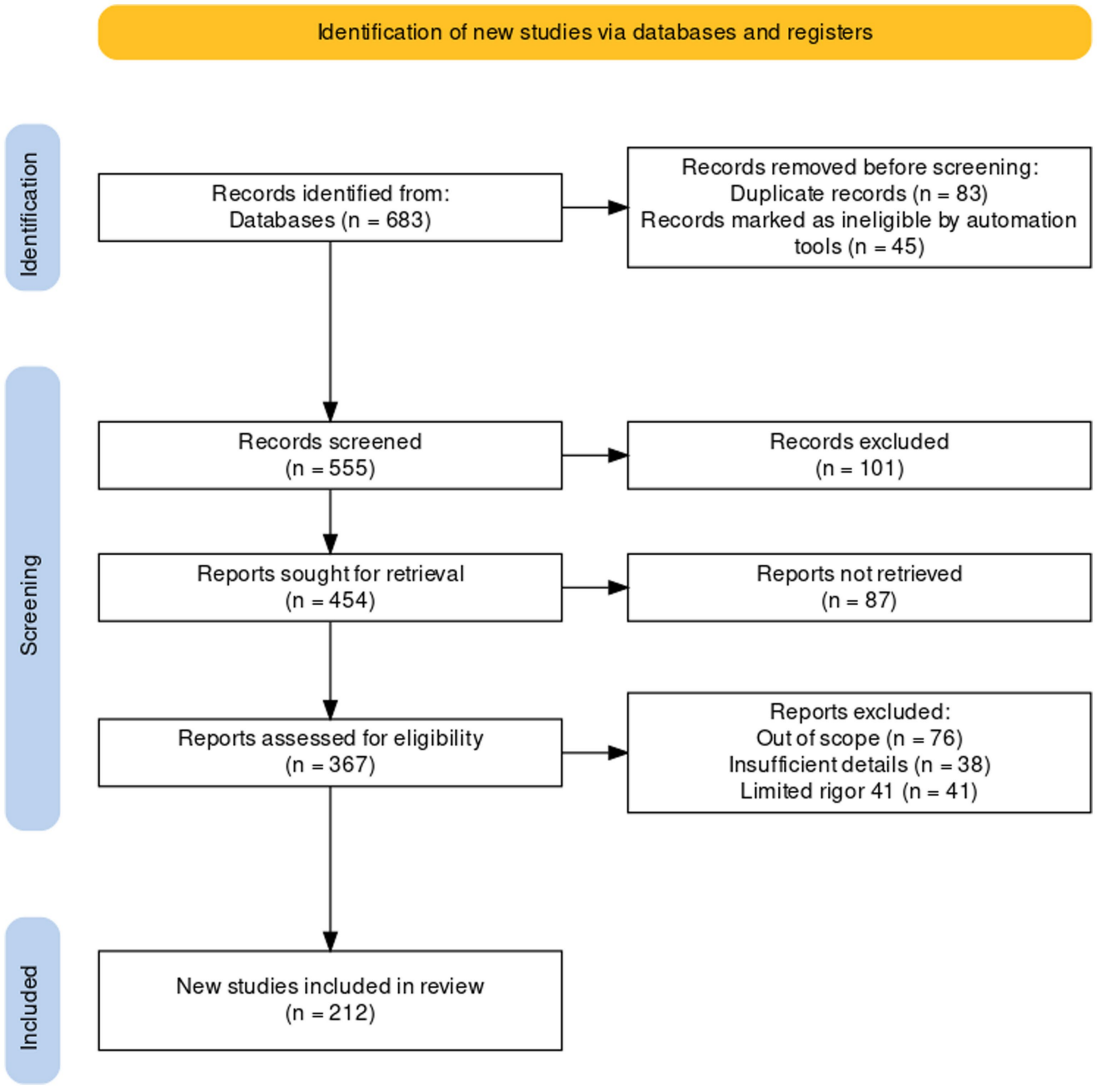
The PRISMA Flow diagram showing the flow of information in the procedure of studies included in this review (Liberati et al., 2009).

A comprehensive literature search was undertaken to explore the legume-nodule-associated endophytic bacteria, unveils their diversity, ecological roles, and benefits for plants. Scientific papers published until the end of March 2024 in literature databases such as Scopus (150), Science-Direct (97) and Google-Scholars (162), Web of Science (174), and others (101) were included. The total number of papers utilized was 683 (Figure 1). To collect this data, we used the combination of several terms and different keywords: nodule endophytes, non-rhizobial endophytes, non-rhizobial bacteria with the term “Diversity,” “novel species,” “sp. nov.” and “Metagenomic”.

After conducting the search, we identified a total of 683 papers that met our inclusion criteria. To screen and select the most relevant papers, we used a PRISMA strategy (systematic and transparent process that involved reading the titles and abstracts of the papers, assessing them for eligibility, and excluding those that did not meet our inclusion criteria). After the screening process, we included 216 studies in this review. More information about the workflow of the strategy of PRISMA is presented as Supplementary material (Supplementary Table S1).

**Figure 2:**
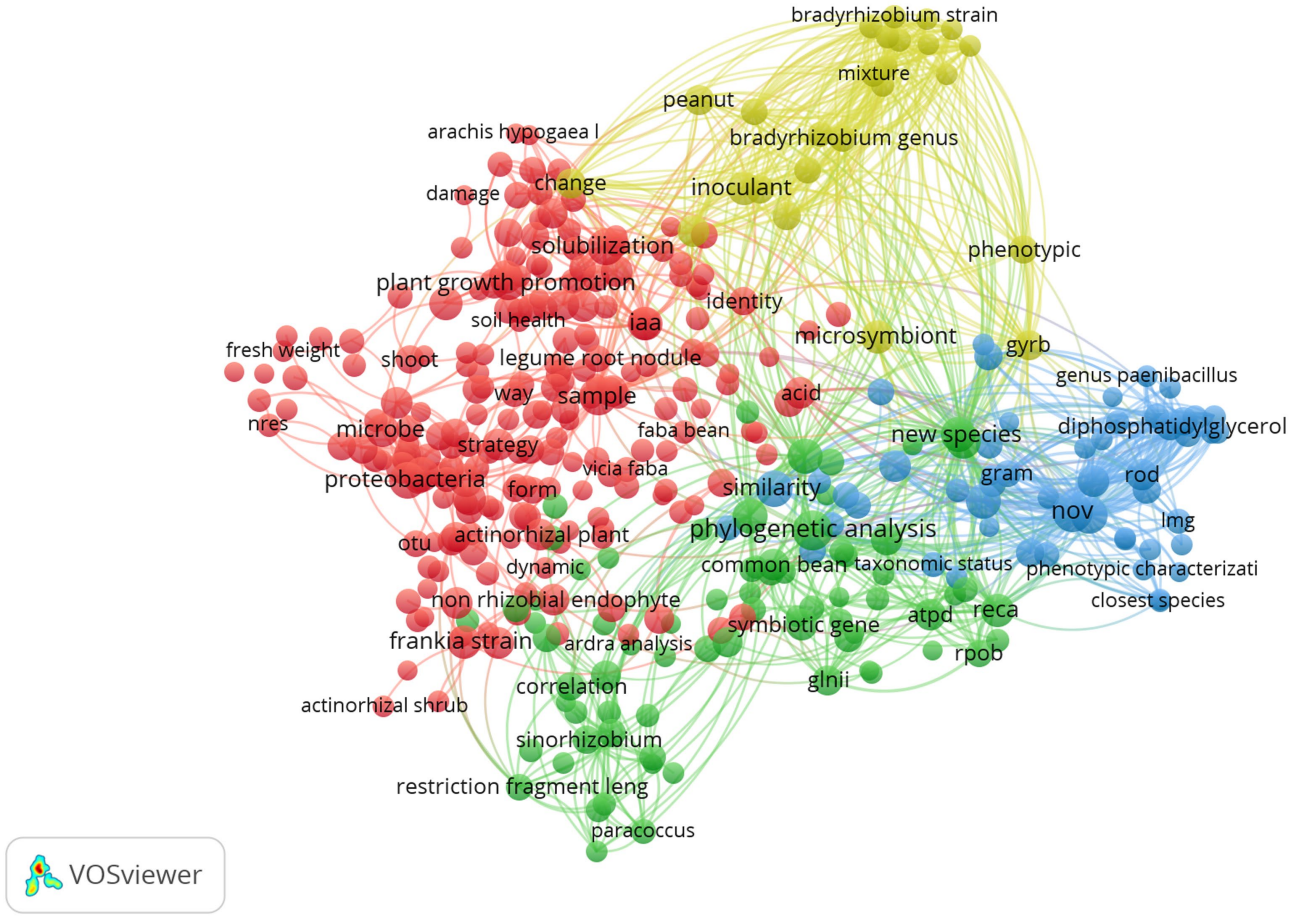
Term clustering map based on the first reference data of systematic review before screening (370 references). Different colors (red, green, blue and yellow) represent the terms belonging to different clusters. The size of the term is based on the number of occurrences. The connecting lines indicate the 100 strongest co-occurrence links between terms (Van Eck and Waltman, 2011).

A vizualisation of the library (683 papers) was presented in Figure 2, which showcases terms with occurrences equal to or exceeding 20, provides a visual representation of the diverse and multidisciplinary nature of research in this topic. The prevalence of terms such as “proteobacteria” “Plant growth promoting,” “solubilization” and “iaa” for the red cluster, and “phylogenetic analysis,” “*sinorhizobium*,” “new species” for the green one. While for the yellow, there is “microsymbiont,” “bradyrhizobium,” and “inoculant.” In the blue cluster, there is a noteworthy prevalence of terms such as “nov,” “similarity,” and “closest species” reflecting the emphasis on understanding the role of non-rhizobial endophytes in the taxonomy and the description of new species.

## 3 Non-symbiotic endophytic bacteria in nodules

### 3.1 Discovery of nodule endophytes

For more than a century, rhizobia belonging to the α-proteobacteria clade were believed to be the exclusive bacteria capable of inducing nodules in legumes, leading to the exclusion of atypical colonies during the screening process on Yeast Mannitol Agar (YMA) medium (Moulin et al., 2001). These authors were the first to identify an atypical symbiotic bacterium belonging to the β-proteobacteria, mainly the genus *Burkholderia*. Later, Peix et al. (2015) identified two atypical bacteria, *Methylobacterium* and *Burkholderia*, capable of nodulating legumes, challenging the traditional view and opening new avenues of inquiry into “non-rhizobial bacteria inducing legume nodules.” Subsequent research uncovered a diverse array of bacteria in legume nodules outside the classic *Rhizobiaceae* family, raising questions about their nitrogen-fixing symbiotic nature (Table 1).

**Table 1:** Characteristics of endophytic bacteria in legume nodules.

Genera/Species	Legumes	Origin	References
*Agrobactérium deltaense*, *A. radiobacter*, and *A. pusense*	*Phaseolus vulgaris*	Spain	Castellano-Hinojosa et al. (2021)
*Acidobacteria*, *Actinobacteria*, *Bacteroidetes*, and *Cyanobacteria*	*Phaseolus vulgaris*	2 substrates (soil and vermiculite)	Barraza et al. (2020)
*Pseudomonas* spp	*Medicago sativa*	Mediterranean origins	Wigley et al. (2017)
*Actinobacteria*, *Firmicutes*, *Bacillus* and *Micromonospora*	*Medicago sativa*	Complex ecosystem	Martínez-Hidalgo et al. (2022)
*Nitrobacter*, *Tardiphaga*, *Bacillus*, *Pseudomonas*, *Flavobacterium*, and *Variovorax*	*Glycine max*	North and central China	Mayhood and Mirza (2021)
*Enterobacter*, *Acinetobacter*, *Pseudomonas*, *Ochrobactrum*, and *Bacillus*	*Glycine max*	Henan Province - China	Zhao et al. (2018)
*Proteobacteria, Firmicutes, Actinobacteria* and *Cyanobacteria*	*Vicia faba*	Northern China	Trabelsi et al. (2017)
*Enterobacter*	*Vicia faba*	Arid region of Morocco ‘Tata-Akka’	Taoufiq et al. (2018)
*Pseudomonas syringae* pv. *pisi*, *Azotobacter chroococcum*, *Rhodococcus erythropolis*	*Pisum sativum*	Aqueous medium for root growth of seedlings in hydroponic conditions	Makarova et al. (2021)
*Ochrobactrum* and *Enterobacter*	*Pisum sativum*	_	Tariq et al. (2014)
*Burkholderia cepacia*	*Cicer arietinum*	Mediterranean origins	Mir et al. (2021)
*Pantoea dispersa* and *Kosakonia oryzae*	*Arachis hypogaea*	_	Preyanga et al. (2021)
*Cupriavidus, Pseudomonas, Bacillus, Acinetobacter, Enterobacter, Roseomonas, Paracoccus, Frondihabitans, Microbacterium, Kocuria, Providencia,,Micrococcus, Staphylococcus*	*Trifolium repense*	Site minier de plomb et de zinc de Guelma, Algérie	Rahal and Chekireb (2021)
*Micromonospora*	*Lupinus angustifolius*	Castile and León/Saelices/Extremadura, Spain	Trujillo et al. (2010)
*Bacillus subtilis Enterobacter tabaci, Bacillus cereus, Bacillus velezensis*	*Lens culinaris*	Saline soil	Jabborova et al. (2021)
*Pseudomonas*, *Pantoea*, *Paenibacillus*, *Brevibacillus*, *Serratia*, *Arthrobacter*, *Burkholderia*	*Mimosa scabrella*	Brazil	Primieri et al. (2016)
*Pseudomonas* spp. *Serratia liquefaciens, Paracoccus sp*	*Cajanus cajan*	Agricultural fields in Vadodara, Gujarat, India	Gosai et al. (2020)
*Enterobacter Pseudomona Stenotrophomonas*	*Vigna unguiculata*	Semi-arid tropical zones of Kenya	Muindi et al. (2021)
*Pseudomonas, Rahnella, Klebsiella, Pantoea, Enterobacter, Xanthomonas, Bacillus, Paenibacillus*	*Lupinus albus*	Tunisia	Ferchichi et al. (2019)
*Pseudomonas putida, Pseudomonas brassicacearum, Pseudomonas frederiksbergensis, Pantoea agglomerans, Serratia proteamaculans, Agrobacterium tumefaciens, Proteamaculans, Bacillus* sp.	*Hedysarum spinosissimum*	Touissit/Sidi Boubker/Oued El Heimer, Oujda, Morocco	Sbabou (2016)
*Pseudomonas* sp.*, Microbacterium* sp.*, Microbacterium* sp.*, Pantoea agglomerans*	*Sulla coronaria*	Sardinia/Italy	Muresu et al. (2019)
*Micromonospora* sp.*, Bacillus* sp.*, Pseudomonas* sp.	*Lotus parviflorus*	Portugal	Soares et al. (2020)
*Enterobacter, Serratia* sp.	*Mimosa pudica*	Humid tropical rainforest of Acandona in Chiapas, Mexico	Sánchez-Cruz et al. (2019)
*Mitsuaria noduli* sp.	*Robinia pseudoacacia*	Lead-zinc mine in China	Fan et al. (2018b)
*Proteobacteria, Sphingobacteria, Enterobacter hormaechi, Stenotrophomonas maltophilia, Enterobacter turicensis*	*Clitoria tematea L.*	_	Aeron et al. (2020)
*Bacillus, Klebsiella, Micromonospora, Pantoea, Pseudomonas, Alphaproteobacteria (Bosea, Devosia, Microviga, Ochrobactrum, Phyllobacterium), Betaproteobacteria (Cupriavidus, Bulkholderia)*	*Chamacus ruthenicus*	Pologne	Kalita et al. (2020)
*Dyadobacter, Flavobacterium, Streptomyces, Sphingobacteria*	*Vigna radiata* (L.) R. Wilczek	field experimental area of NIBGE, Faisalabad, Pakistan	Hakim et al. (2018)
*Actinobacteria, Bacteroidetes, Proteobacteria, Chioroflexi, Firmicutes, Acidobacteria, Gemmatimonadetes, Nitrospirae*	*Vigna radiata*	from the field. Gujarat, India	Iyer and Rajkumar (2017)
*Enterobacter, Chryseobacterium, Sphingobacterium*	*Vigna unguiculata*	Embrapa Semiárido, Northeast of Brazil	Leite et al. (2017)
*Agrobacterium, Arthrobacter, Acinetobacter, Bacillus, Bosea, Enterobacter, Micromonospora, Mycobacterium, Paenibacillus, Pseudomonas,* and *Stenotrophomonas*	_	_	Velázquez et al. (2013)
*Agrobacterium*	common bean (*Phaseolus vulgaris* L.)	in a Northern Tunisian field	Mnasri et al. (2012)
*Cohnella, Leifsonia, Luteibacter, Novosphingobium, Paenibacillus,* and *Pantoea*	*Macroptilium atropurpureum*	the Quadrilátero Ferrífero region, MG, Brazil,	Oliveira Silva et al. (2020)
*Arthrobacter, Bacillus, Bradyrhizobium, Burkholderia, Dyella, Methylobacterium, Microbacterium, Rhizobium,* and *Staphylococcus*	*Lespedeza sp*	Chungbuk National University, Cheongju, South Korea.	Palaniappan et al. (2010)
*Bosea thiooxidans*	*Spartocytisus supranubius*	in soils of the Teide National Park (Tenerife, Spain).	Pulido-Suárez et al. (2022)
*Micromonospora lupini, Micromonospora saelicesensis*	*Lupinus angustifolius*	_	Trujillo et al. (2007)
*Micromonospora pisi*	*Pisum sativum*	_	Garcia et al. (2010)
*Pseudonocardia acaciae*	*Acacia auriculiformis*	_	Duangmal et al. (2009)
*Phyllobacterium, Starkeya* and *Pseudomonas.*	*Lotus* sp.	Tunisian arid soils	Rejii et al. (2014)
*PaeniBacillus, Bacillus, Ralstonia, Cohnella, Lysinibacillus, Sphingobium*	*Acacia disparrima and A. leiocalyx*	Toohey Forest, a dry sclerophyll subtropical forest located in Brisbane, southeast Queensland, Australia.	Reverchon et al. (2020)
*Achromobacter, Agrobacterium, Burkholderia, Cronobacter, Enterobacter, Mesorhizobium, Novosphingobium, Pantoea, Pseudomonas, Rahnella, Rhizobium, Serratia,* and *Variovorax*	*Crotalaria* spp.*, Indigofera* spp.*, and Erythrina brucei*	Ethiopia	Aserse et al. (2013)
*Agrobacterium tumefaciens complex* and *A. radiobacter,*	*(Phaseolus vulgaris L.)*	in Ethiopia	Aserse et al. (2012)
*Phyllobacterium, Arthrobacter, Variovorax* and *Pseudomonas*	*Sulla pallida* and *Sulla capitata.*	Algeria and Tunisia	Beghalem et al. (2017)
*Staphylococcus, Bacillus, PaeniBacillus, Enterobacter, Stenotrophomonas*	*Erythrina brucei*	different land use types in Ethiopia	Berza et al. (2021)
*Enterobacter cloacae, Arthrobacter luteolus, Microbacterium, Curtobacterium, Microbacterium ginsengisoli, Bacillus*	*Colophospermum mopane*	Kunene region of Namibia	Burbano et al. (2015)
*Bacillus, Burkholderia, Enterobacter, Franconibacter, Pseudomonas* and *Williamsia*	*Phaseolus lunatus*	soils from Piauí State, Northeast Brazil	Chibeba et al. (2020)
*Agrobacterium* sp. *10C2*	*Phaseolus vulgaris.*	Cap Bon in northeast Tunisia.	Chihaoui et al. (2015)
*Bacillus megaterium*	*Medicago polymorpha*	Ciudad Real, Spain	Chinnaswamy et al. (2018)
*Actinoplanes, Aeromicrobium, Arthrobacter, Brevibacterium, Corynebacterium, Curtobacterium, Kocuria, Leifsonia, Microbacterium, Moraxella, Mycobacterium, Oerskovia, Plantibacter, Promicromonospora, Rhodococcus, Schumanella and Streptomyces, Bacillus, Brevibacillus, Cohnella, Exigobacterium, Lysinibacillus, Paenibacillus and Staphylococcus, Ancylobacter, Bosea, Caulobacter, Inquilinus, Novosphingobium, Paracoccus, Phyllobacterium and Sphingomonas, Herbaspirillum, Massilia, Roseateles and Variovorax, Acinetobacter, Buttiauxella, Enhydrobacter, Erwinia, Pantoea, Pseudomonas, Rahnella, Stenotrophomonas* and *Xanthomonas*	indigenous legumes	Flanders (Belgium)	De Meyer et al. (2015)
*Bacillus pumilus Qtx-10* and *Streptomyces bottropensis Gt-10*	*Sphaerophysa salsula*	northwestern China	Deng et al. (2020)
*Aracoccus, Sphingomonas, Inquilinus, Pseudomonas, Serratia, Mycobacterium, Nocardia, Streptomyces, Paenibacillus, Brevibacillus, Staphylococcus, Lysinibacillus* and *Bacillus*	*Sphaerophysa salsula*	Loess Plateau in China	Deng et al. (2011)
*Agrobacterium*	*Phaseolus vulgaris L.*	Skhirat, Berkane, Sidi Allal Tazi region and Gharb	El Attar et al. (2019)
*A. radiobacter HZ6*	*Robinia pseudoacacia*	Shaanxi Province, China.	Fan et al. (2018a)
*Pseudomonas* sp.	*Medicago* spp.	Huelva, Spain	Flores-Duarte et al. (2022a)
*Variovorax paradoxus S110T* and *Variovorax gossypii JM-310 T*	*Medicago sativa*	_	Flores-Duarte et al. (2022b)
*Acinetobacter, Microbacterium, Pseudomonas*	*mung bean (Vigna radiata L.)*	Pakistan	Hakim et al. (2020)
*Enterobacter sp (BCH13, BCH2), Pseudomonas sp (BCH16, BCH24),* and *Serratia sp (BCH10)*	*Sulla flexuosa*	Northeastern Morocco	Hamane et al. (2023)
*Bacillus, Citrobacter, Enterobacter, Novosphingobium, Paenibacillus, Pantoea*	*Ormosia macrocalyx*	Nacajuca, Tabasco, Mexico.	Hernández-Hernández et al. (2018)
*Bacillus, Pseudomonas, Microbacterium, Klebsiella Stenotrophomonas, Streptomyces*	*Vachellia tortilis* subsp*. raddiana*	Guelmim Morocco	Hnini et al. (2023)
*Phyllobacterium* and *Devosia.*	*Acacia salicina and A. stenophylla*	south-eastern Australia	Hoque et al. (2011)
*Bacillus, Enterobacter, Herbaspirillum, Mistsuaria, Pantoea, Pseudomonas*	*Peanut (Arachis hypogaea L.)*	indigenous South American legume	Hossain et al. (2023)
*Acinetobacter calcoaceticus, Arthrobacter* sp.*, Bacillus pseudomycoides, Bacillus* sp.*, Microbacterium hydrocarbonoxydans, Niallia* sp.*, Paenarthrobacter nitroguajacolicus, Paenarthrobacter* sp.*, Peribacillus frigoritolerans, Peribacillus* sp.*, Plantibacter flavus, Priestia aryabhattai, Priestia megaterium, Pseudomonas koreensis, Psychrobacillus* sp.*, Rossellomorea marisflavi*	*Lens culinaris*	*Southern Italy*	Brescia et al. (2023)
*Pseudomonas,Enterobacter* and *Klebsiella*	*(Arachis hypogaea L.)*	Argentina	Ibañez et al. (2014)
*Agrobacterium, Phyllobacterium*	*Acacia Saligna*	Morocco	Lebrazi et al. (2023)
*Sphingomonas, Paracoccus, Mycobacterium, Paenibacillus, Cohnella, Sporosarcina, Bacillus, Staphylococcus, Brevibacterium, Xenophilus, Erwinia, Leclercia, Acinetobacter, and Pseudomonas*	*C. jubata and O. ochrocephala*	china	Xu et al. (2014)
*Pantoea Pseudomonas Stenotrophomonas Serratia* spp. *Brevibacterium sp Bacillus sp*	*Cicer arietinum*	Morocco	Benjelloun et al. (2019)
*Serratia marcescens*	*Cicer arietinum*	Thal desert	Zaheer et al. (2016)
*Bacillus subtilis NANEB1* and *Paenibacillus taichungensis TNEB6*	*Vigna mungo*	_	Srt et al. (2019)
*Serratia plymuthica 33GS* and *Serratia* sp. *R6*	*Lens culinaris*	India	Debnath et al. (2023)
*Rhodococcus*	*Lotus corniculatus and Anthyllis vulneraria*	Sweden	Ampomah and Huss-Danell (2011)
*Arthrobacter protophormiae*	*Pisum sativum*	India	Barnawal et al. (2014)
*Chryseobacterium, Bacillus, Microbacterium, Agrobacterium, Escherichia, Delftia, Pelomonas, Sphingomonas,* and *Staphylococcus.*	*Vigna unguiculata*	_	da Silva et al. (2023)
*Paenibacillus farraposensis*	*Arachis villosa*	Uruguay	Roldán et al. (2022)
*Paenibacillus* sp.	*Arachis villosa*	_	Costa et al. (2022)
*Amycolatopsis* spp.	*Soybean*	Japan	Kataoka et al. (2022)
*Rahnella aquatilis* and *Serratia plymuthica*	*Vicia fava and Pisum sativum*	Italy	Novello et al. (2022)
*Bacillus cereus MY5, Ralstonia pickettii MY1* and *Lactococcus lactis MY3*	*Mimosa pudica*	Kerala, India.	Ravunni and Yusuf, 2022
*Bacillus zanthoxyli, TG47 as Phyllobacterium zundukense* and *TG68 as Pantoea agglomerans.*	*Lespedeza daurica*	China	Kai-yuan et al. (2023)
*Bacillus megaterium BMN1*	*Medicago sativa L.*	_	Khalifa and Almalki, 2015
*Enterobacter cloacae MSR1*	*Medicago sativa L.*	_	Khalifa et al. (2016)
*Proteobacteria, Actinobacteria, Firmicutes* and *Bacteroidetes*	*soybean*	China	Xiao et al. (2017)
*Enterobacter* spp.	*Abrus precatorius L.*	_	Kumar Ghosh et al. (2015)
*Tardiphaga* and *Synechococcus*	*Acacia longifolia*	Mira, Aveiro, Northern Coastal Portugal	Jesus et al. (2023)
*Pantoea agglomerans (CPHN2), Bacillus cereus strain (CPHN4), Bacillus sonorensis strain (CPHN12), Bacillus subtilis strain (CPHR3), Pseudomonas chlororaphis strain (PHN9), Ornithinibacillus* sp. *(PHN14), Ochrobactrum* sp. *(PHR6).*	*Cicer arietinum and Pisum sativum:*	Haryana state, India	Maheshwari et al. (2019)
*Micromonospora*	*Medicago sativa*	Castilla y Leo ´n (Spain).	Martínez-Hidalgo et al. (2014)
*Shinella, Rahnella, Bacillus, Pantoea, Pseudomonas, Xanthomonas*	*Vicia faba*	north and the south of Tunisia	Saïdi et al. (2013)
*Agrobacterium* and *Shinella.*	*Vicia faba, Cicer arietinum and Phaseolus vulgaris*	different soils in Tunisia	Saïdi et al. (2011)
*Bacillus sp*	*Cicer arietinum*	Northern India	Saini et al. (2015)
*Bacillus, Curtobacterium, Phyllobacterium, Pseudomonas*	(*Trifolium pratense* L.)	Prince Edward Island, Canada	Sturz et al. (1997)
*Chitinophaga, Bosea thiooxidans, Caulobacter, Agrobacterium, Geobacillus, Pseudomonas, Inquilinus, Halomonas*	*Vigna unguiculata*	California	Sun et al. (2015)
*Sphingomonadaceae, Shinella, Aurantimonas, Hyphomicrobium, Comamonas, Hydrogenophaga, Variovorax, Xenophilus, Acidovorax, Acidovorax, Delftia, Herbaspirillum, Methylibium, Rhizobacter, Roseateles, Pelomonas, Aquabacterium, Acidovorax, Pseudomonas, Pseudoxanthomonas*	*soybean*	China	Zhang et al. (2011)
*Enterobacter, Acinetobacter, Pseudomonas, Ochrobactrum, and Bacillus.*	*Glycine max*	Henan Province, China	Zhao et al. (2018)
*Agromyces, Microbacterium Retama taetam, Ononis natrix, Argyrolobium uniflorum*	*Astragalus armatus*	_	Zakhia et al. (2006)
*Staphylococcus hominis 7E, Streptomyces* sp. *11E, Bacillus* sp. *13E, Acinetobacter* sp. *19E,*	*Vigna radiata*	Khuzestan	Bakhtiyarifar et al. (2021)
*Staphylococcus hominis 9E, Bacillus endophyticus 14E, Brevundimonas* sp. *16E* and *Kocuria* sp. *26E*	*Glycine max*	Khuzestan	Bakhtiyarifar et al. (2021)
*Novosphingobium, Arthrobacter, Bacillus, Caulobacter, Pseudomonas, Flavobacterium, Niastella, Streptomyces, Klebsiella,, Chryseobacterium, Variovorax, Stenotrophomonas, Pantoea,*	*Glycine max*	São Paulo, southeast Brazil.	Bender et al. (2022)
*Taibailla, Inquillinus, Cupriaavidus, Pantoea, Sphingopyxis, chitinophaga, sphingomonas, Canobacterium, Lactococcus, Pelomonas, Ralstonia, Sporosarcina, Acinetobacter, Stenotrophomonas, Chryseobacterium, enterobacter, Flavobecterium, Sphingobacterium, Microbacterium, Pseudomonas, Lysobacter.*	*Vigna radiata*	Pakistan	Hakim et al. (2020)
*Tardiphaga, Synechococcus, Geminocystis, Mycoplasma, Stanieria, Actinomyces, Massilia, Acaryochloris, Oscillatoria,* and *Rhodopseudomonas.*	*Acacia longifolia*	Aveiro, Northern Coastal Portugal	Jesus et al. (2023)
*Achromobacter, Rahnella, Serratia, Pantoea, Pseudomonas, Enterobacter* and *Paenibacillus*	*Vicia faba, Glycine max*	Latvia	Klūga et al. (2023)
*Pseudomonas, Novosphingobium, sphingomonas, shinella, variovorax, acidovorax, pseudarcicella, bosea, bacillus, tardiphaga, Nitrobacter*	*Glycine max*	Clarkton Missouri, United States	McElveen (2019)
*Ohtaekwangia Polaromonas Brevundimonas Rhizobacter Dyadobacter Flavobacterium Opitutus Staphylococcus Enhydrobacter Dongia Tahibacter Pelomonas*	*Lablab purpureus*	Clarkton Missouri, United States	McElveen (2019)
*Bacillus tequilensis, Pantoea dispersa, Paenibacillus illionoisensis, Bacillus altitudinus* and *Kosakonia oryzendophytica.*	*Arachis hypogaea*	Tamil Nadu, India	Preyanga et al. (2021)
*Pantoea vagans, Luteibacter rhizovicinus, Klebsiella oxytoca, Pseudomonas azotoformans, Pantoea rodasi,* and *Achromobacter spanius*	*Genista cinerea*	Arid zones of North Africa	Dekak et al. (2020)

The discoveries disclosed that legume nodules in the field host not only symbiotic rhizobia but also non-symbiotic endophytes, complicating the immune control in symbiotic organs (Dudeja et al., 2012). Some studies indicated a prevalence of non-symbiotic endophytes over rhizobia in certain plant nodules, suggesting specificity in the nodular microbiome, in addition to the established specificity between rhizobia and their host plants. Moreover the plant’s immunity appears to play a role in recognizing these non-rhizobial endophytes (Berrabah et al., 2019).

Research by Youseif et al. (2021) and others reveals diverse bacterial endophytes in legume nodules. Genera like *Bacillus, Pseudomonas,* and *Agrobacterium* coexist with rhizobia. Other studies identified genera such as *Arthrobacter* and *Acinetobacter* as nodule endophytes (Velázquez et al., 2013). Legumes like cowpea host a variety of endophytes including *Cupriavidus* and *Providencia* (Leite et al., 2017). Indigenous legumes in Flanders also show a rich diversity of endophytes, including *Agrobacterium* and *Klebsiella* (De Meyer et al., 2015).

Other studies concerning root nodules of different legume species, have identified various genera such as Arthrobacter, Microbacterium, Rhodococcus, Sphingomonas, Bacillus, Cohnella, Pseudomonas, Herbaspirillum, Rahnella, Enterobacter, Agrobacterium, Burkholderia, Pantoea, Paenibacillus, Klebsiella, Endobacter, Microbacterium, Curtobacterium, Xenophilus, Erwinia, Leclercia, Gluconacetobacter, Variovorax, Staphylococcus, Methylobacterium, Paraburkholderia, Brevibacterium, Microvirga, Strepromyces, Micromonospora, and Hyphomicrobium (Sturz et al., 1997; Valverde, 2003; Mantelin et al., 2006; Zakhia et al., 2006; Zurdo-Piñeiro et al., 2007; Palaniappan et al., 2010; Aserse et al., 2013; Velázquez et al., 2013; Xu et al., 2014; Dhole et al., 2016; Zaheer et al., 2016; Martínez-Hidalgo and Hirsch, 2017; Xiao et al., 2017; Zhang et al., 2018; El Attar et al., 2019; Raja and Uthandi, 2019; Deng et al., 2020).

Recent studies have further highlighted the complexity of root nodules, emphasizing the presence of non rhizobial root nodule endophytes (NRE) with diverse roles within the host. These NREs enter nodules through infection threads containing rhizobia and colonize the inner regions of root nodules (Pandya et al., 2013; Zgadzaj et al., 2015). While these bacteria do not independently induce nodules, they enhance nodule formation when co-inoculated with suitable rhizobia, displaying various plant growth-promoting properties (Li et al., 2012).

It is interesting to highlight the case of some *Agrobacterium* strains, including those isolated from common bean root nodules and other legumes, that were reported as having the capacity to nodulate legume plants (De Lajudie et al., 1999; Verástegui-Valdés et al., 2014; El Attar et al., 2019). This nodulation ability may be attributed to the acquisition of symbiotic genes via lateral gene transfer, as suggested in various studies (Moulin et al., 2004; García-Fraile et al., 2010). Supporting this idea, some *Agrobacterium* strains were found to carry symbiosis-specific genes (e.g., *nifH* and *nodA*), similar to those present in well-known rhizobia legume symbionts (Cummings, 2009; Rincón-Rosales et al., 2009; Youseif et al., 2014). These reports highlight the rich diversity and dynamic interactions of the nodule endophytes, and their contribution to the intricate and multifaceted nature of the legume-nodule ecosystem.

### 3.2 Prevalence of endophytes in legume nodules

**Figure 3:**
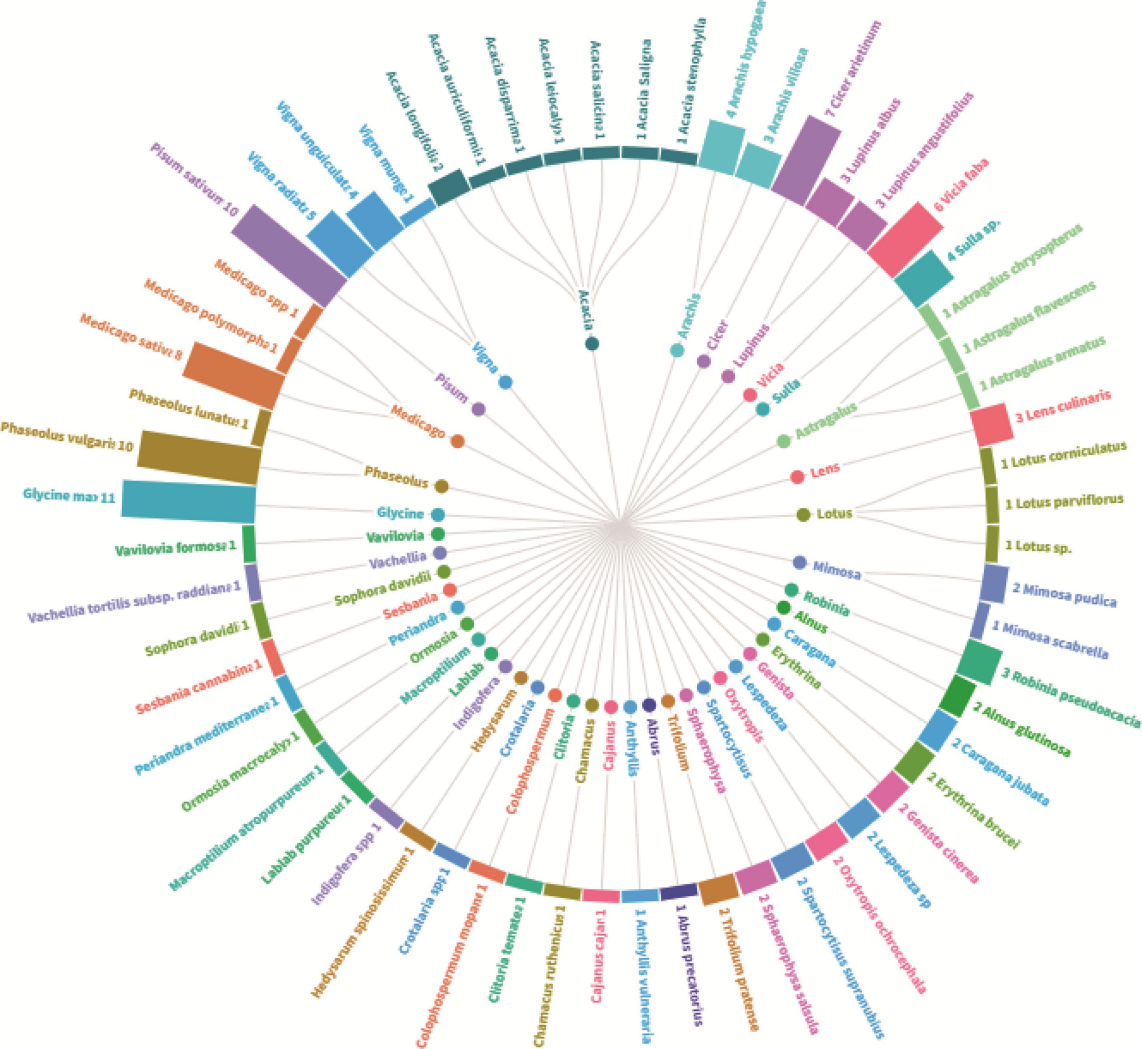
Plant diversity and research focus–an overview of the number of studies on the level of plant species for nodule endophyte bacteria.

To provide an in-depth understanding of the main explored plant species, Figure 3 encapsulates a comprehensive overview of the legume species under investigation, highlighting the predominance of the species *Glycine max* which stands out with 11 associated studies. At the same level, *Phaseolus vulgaris* and *Pisum sativum* with 10 studies for each plant. Other frequently scrutinized genera encompass *Medicago, Cicer, Vicia, Vigna*; that were the subject of 5 to 8 studies. The genus *Acacia* is notably diverse, with studies spanning multiple species. In the other side, certain species, such as those belonging to *Lotus* and *Astragalus* are comparatively underexplored, each featuring in only one study. The dataset reflects a broad spectrum of leguminous plants, underscoring their significance in research. Another important aspect concerns the botanical diversity of the studied species that ranged from trees like *Robinia pseudoacacia* to shrubs like *Spartocytisus supranubius*, through food and fodder legume species. Some species, including *Lupinus angustifolius* and *Erythrina brucei* garner repeated attention, indicating sustained research interest. In essence, Figure 3 offers a nuanced portrayal of botanical studies, emphasizing both the diversity of plants investigated and the varying degrees of research focus across different genera and species.

**Figure 4:**
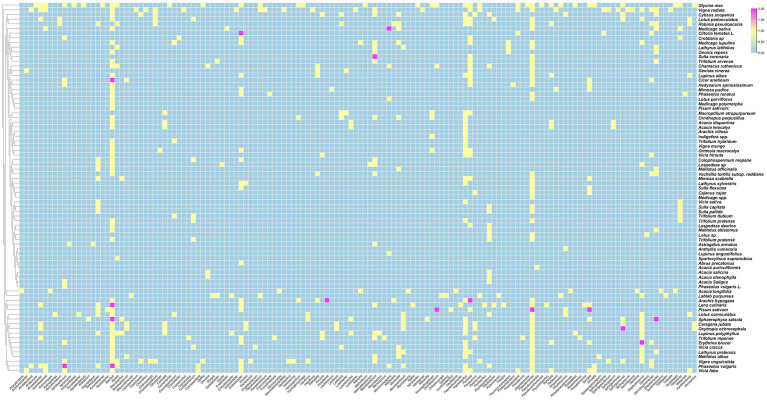
Heatmap representing the main Plant species in this study along with the associated nodule endophyte genera.

**Figure 5:**
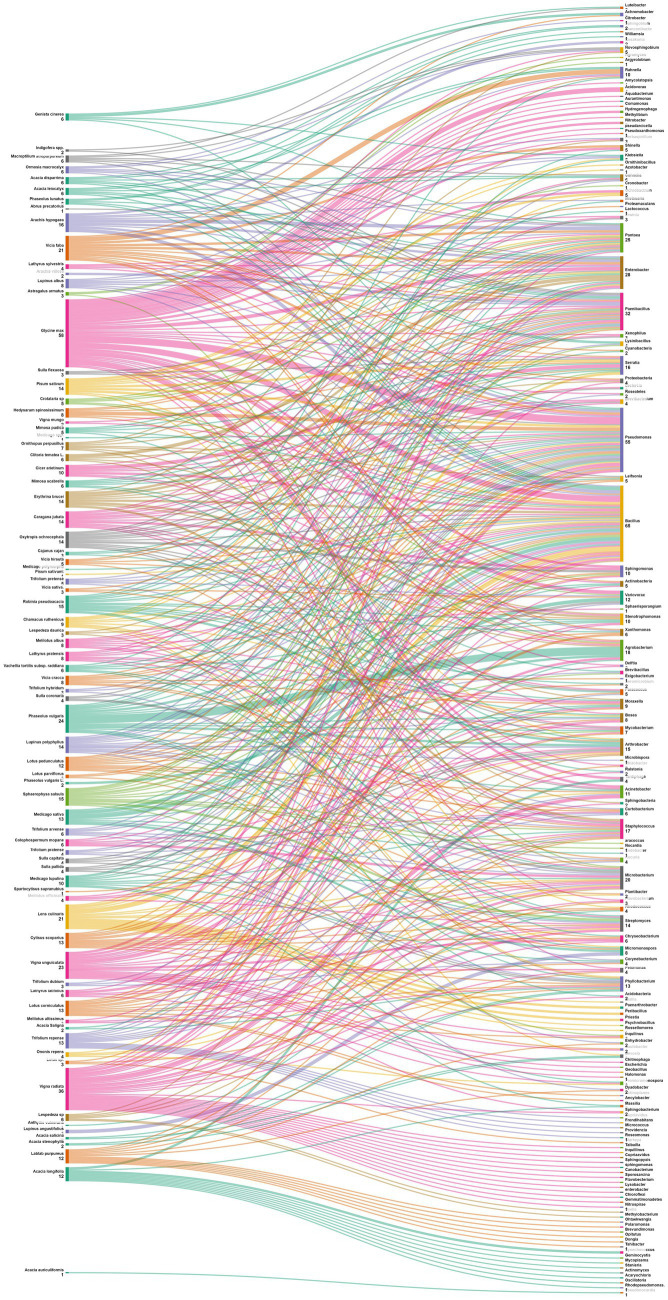
Sankey diagram representing the main Plant species in this study along with the associated nodule endophyte genera.

The data analysis performed showed that the most abundant endophytic bacteria were *Bacillus* (65) and *Pseudomonas* (55) followed by *Paenibacillus* (32), *Enterobacter* (28), *Pantoea* (25), *Agrobacterium* and *Microbacterium* (Figures 4, 5). While for plants, *Glycine max* was the species that host the majority of nodule endophytic bacteria followed by *Phaseolus vulgaris*, *Vigna unguiculata* and *Lens culinaris* (Figure 4). Additionally, the clustering established in Figure 5, reveals a result that aligned with the previous one, *Bacillus* and *Pseudomonas* being the most abundant nodule endophytic bacteria in the whole collection followed by *Paenibacillus, Agrobacterium* and *Enterobacter*.

Legume nodules, that are intricate habitats for diverse bacterial communities, exhibit a prevalence of specific genera dominated by strains affiliated with *Pseudomonas* and *Bacillus* (Hnini et al., 2023). Similar results were reported earlier by Kumar et al. (2013), who isolated a substantial number of Gram-positive bacterial endophytes from legume nodules, spanning chickpea, field pea, and lucerne. Moreover *Bacillus* emerges as a the predominant genus in nodules of various legumes, including chickpea (Joseph et al., 2007), pigeon pea (Rajendran et al., 2008), *Lespedeza* sp. (Palaniappan et al., 2010), peanut (Wang et al., 2013), and soybean (Zhao et al., 2018). It is followed by *Pseudomonas* strains, that occupy the second position in nodules of peanut, soybean and bean (Aserse et al., 2013; Wang et al., 2013; Dinić et al., 2015; Zhao et al., 2018).

The elucidation of mechanisms governing nodule colonization by non-rhizobial endophytes (NREs) represents a critical frontier in understanding legume-microbe interactions (Martínez-Hidalgo and Hirsch, 2017). While immunofluorescence microscopy has confirmed the presence of non-rhizobia within nodules (Muresu et al., 2008), the intricacies of NRE entry into the symbiosome during nodule formation remain elusive. This ambiguity underscores the need for further investigation into the molecular signaling pathways facilitating NRE colonization. Ibáñez et al. (2009) and Zgadzaj et al. (2015) have observed the absence of *nodC* gene among cultured NRE isolates, suggesting alternative mechanisms for NRE entry that are different from those implicated in rhizobial symbiosis initiation. The absence of nod genes in NRE isolates implies a potential reliance on rhizobia-initiated infection threads or entry through root cracks (Giraud et al., 2007), necessitating comprehensive exploration of NRE nodule entry strategies.

Furthermore, recent research conducted by Brown et al. (2020) demonstrated that host genetic factors significantly influence the composition of nodule communities in the model legume *Medicago truncatula.* Through a common garden experiment and sequencing analyses, they revealed that while soil origin shapes rhizosphere communities, internal plant compartments—such as the root endosphere and nodules—are predominantly governed by host genetics. These insights highlight the intricate specificity of interactions between legumes and nodular endophytic bacteria, prompting exploration into the underlying mechanisms driving host selection of these bacteria across diverse environmental conditions. Additionally, the makeup of the nodular microbiota is not solely influenced by the choice of culture media but is also shaped by a range of environmental variables. Factors such as soil type, cultivation practices, and isolation techniques, including the use of wild nodules and trap plants, all play pivotal roles in determining the composition of nodular microbiota. Consequently, gaining a deeper understanding of the molecular and genetic factors that govern legume-nodular endophytic bacteria associations is crucial for comprehending their ecological importance and for devising targeted agricultural strategies.

In parallel, recent investigations by Tokgöz et al. (2020), have revealed the remarkable capacity of NREs to suppress plant pathogens, by discovering diverse bacteria within soybean nodules and identified several non-rhizobial isolates capable of inhibiting plant pathogens like *Clavibacter michiganensis* subsp*. michiganensis* (Cmm) and *Pseudomonas syringae* pv*. tomato* (Pst). By extratcing bacterial metabolites from selected isolates and found a Proteus species to be promising in suppressing pathogens. The findings of Tokgöz et al. (2020), not only highlight the potential of NREs in enhancing legume resilience under adverse environmental conditions but also underscore the intricate network of microbial interactions within legume nodules. This convergence of research underscores the multifaceted nature of legume-microbe associations, necessitating interdisciplinary approaches to unravel their complexities and harness their potential for sustainable agricultural practices.

Diversity of endophytic relationships: patterns of nodule endophyte associations with the subfamily of Papilionoidea

**Figure 6:**
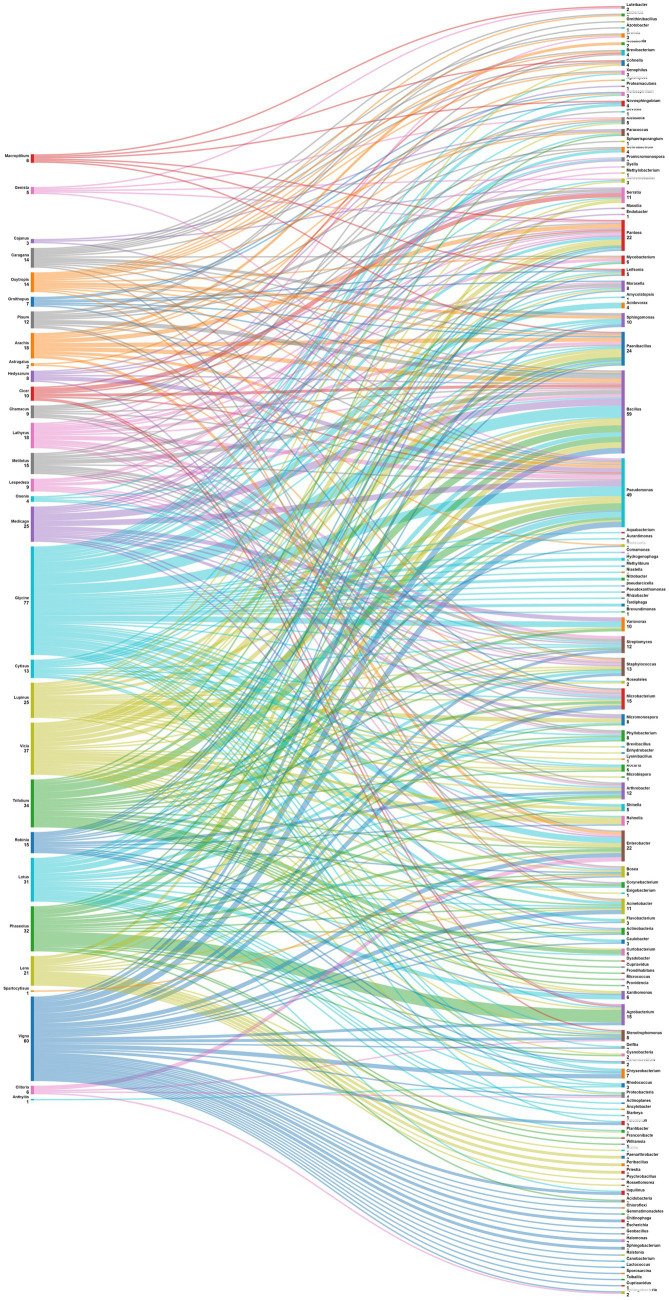
Sankey diagram representing the main plant genera (of the subfamily of *Papilionoidea*) in this study along with the associated nodule endophyte genera.

One of the objectives set for this analysis is to delve into the intricate relationships between legume plants and their associated nodule endophytes, shedding light on the diverse prevalence patterns across various plant genera. The data in Figure 6 suggest a diverse and specific prevalence pattern among plant genera and nodule endophyte genera. Numerous legume genera exhibit associations with specific endophytes, indicating a nuanced symbiotic relationship. For example, *Phaseolus* nodules showed a preference for *Agrobacterium, Acidobacteria, Actinobacteria* and *Cyanobacteria*, while those of *Medicago* contained mostly *Actinobacteria, Bacillus, Micromonospora, Pseudomonas,* and *Endobacter*. On the other hand, *Glycine* nodules displayed a varied pattern, composed by *Nitrobacter, Tardiphaga, Bacillus, Pseudomonas, Flavobacterium,* Var*iovorax, Enterobacter, Acinetobacter, Ochrobactrum,* and *Agrobacterium*. Other legume genera, including *Vicia, Pisum, Cicer, Arachis, Trifolium, Lupinus, Lens, Cajanus, Vigna, Hedysarum, Genista, Robinia, Clitoria, Chamacus,* and *Macroptilium,* exhibited unique associations with diverse nodule endophyte genera.

**Figure 7:**
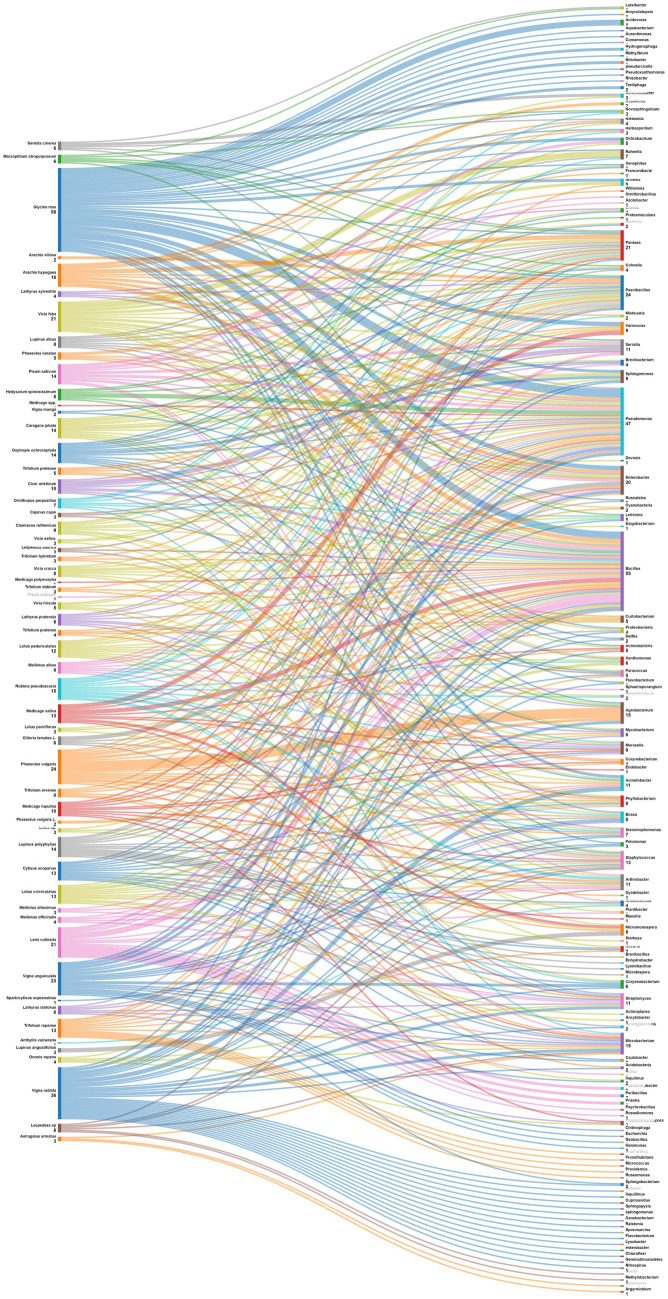
Sankey diagram representing the main plant species (of the subfamily of *Papilionoidea*) in this study along with the associated nodule endophyte genera.

Data were also analyzed at the level of plant species (Figure 7). *Phaseolus vulgaris*, the most studied species is associated with Agrobacterium, Arthrobacter, Acidobacteria, Actinobacteria, Cyanobacteria, Phyllobacterium, Shinella, Acinetobacter, Bosea, Micromonospora, Mycobacterium, Paenibacillus and Stenotrophomonas. For its side, *Medicago sativa* nodules displayed diverse endophytes such as Actinobacteria, Bacillus, and Pseudomonas; while those of *Glycine max* exhibited a different pattern, including Nitrobacter, Tardiphaga, Bacillus, and Pseudomonas. Other leguminous species like *Vicia faba*, *Pisum sativum* and *Cicer arietinum* also demonstrated a variety of associated endophytes. This diversity of patterns underscores the complex nature of plant-endophyte interactions. Understanding these patterns can have implications for agricultural practices, potentially allowing for targeted inoculation with specific endophytes to enhance plant growth, nutrient uptake, and overall health in different legume crops.

Diversity of endophytic relationships: Patterns of nodule endophyte associations with the subfamilies of Mimosoideae, Faboideae, Hedysareae and Caesalpinioideae

**Figure 8:**
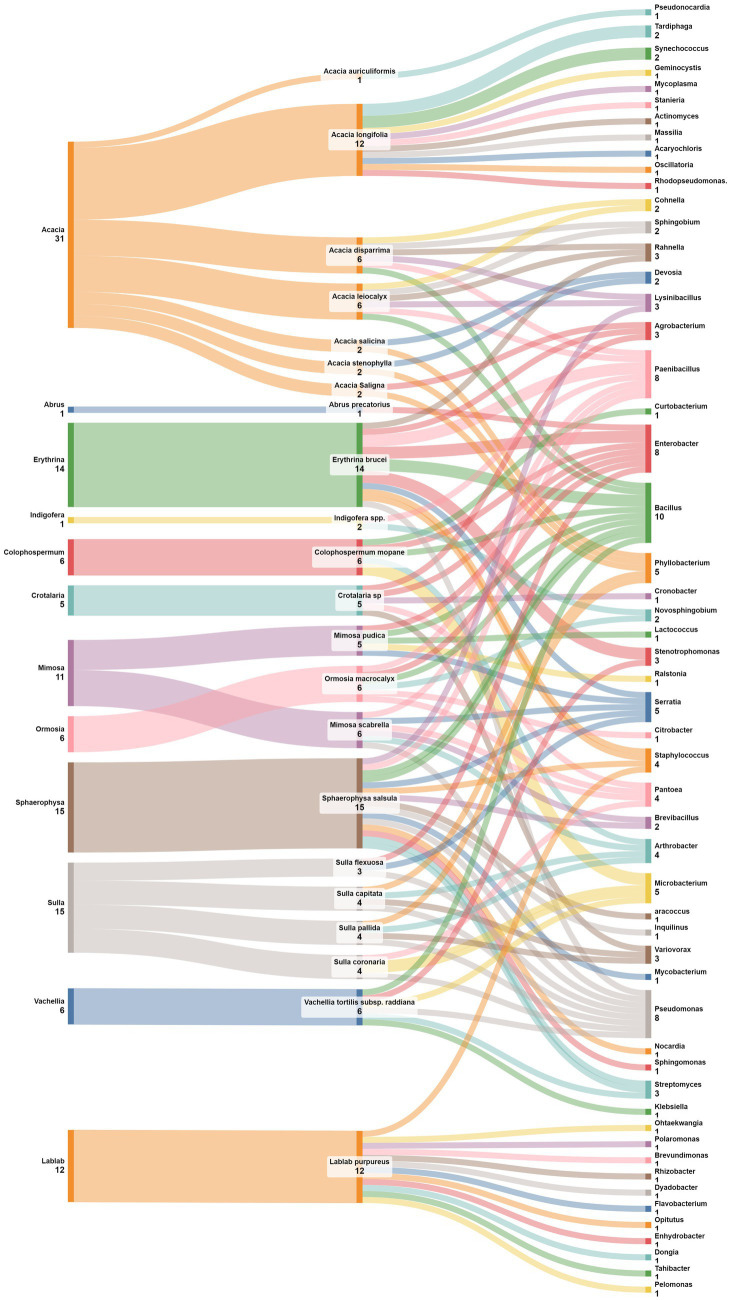
Sankey diagram representing the main plant genera and species (of the subfamilies of *Mimosoideae*, *Faboideae*, *Hedysareae*, and *Caesalpinioideae*) in this study along with the associated nodule endophyte genera.

In a similar manner to the previous findings, Figure 8 unravels distinctive patterns in the association between plant genera belonging to four legumes subfamilies and nodule endophyte genera. Notably, *Crotalaria* is linked to *Enterobacter, Pantoea, Agrobacterium, Cronobacter,* and Var*iovorax.* The legume genus *Indigofera*, in contrast, exhibited a preference for *PaeniBacillus* and *Novosphingobium* while *Erythrina* demonstrated a diverse set of associations with *Pseudomonas, Rahnella, Serratia, Agrobacterium, Staphylococcus, Bacillus, PaeniBacillus, Enterobacter,* and *Stenotrophomonas*. The genus *Colophospermum* is associated with *Enterobacter, Arthrobacter, Microbacterium,* and *Bacillus;* while *Sphaerophysa* showed a preference for *Bacillus* and *Streptomyces. Ormosia* legume genus exhibited associations with *Bacillus, Citrobacter, Enterobacter*, *Novosphingobium, Paenibacillus,* and *Pantoea* while the genus *Vavilovia* is linked to *Bosea*, and the genus *Abrus* is associated with *Enterobacter*. The genus *Periandra* showed a preference for *Paenibacillus,* and *Mimosa* demonstrated a diversified pattern containing *Pseudomonas, Pantoea, Paenibacillus, Brevibacillus, Serratia, Arthrobacter, Burkholderia,* and *Enterobacter*. Similarly, *Sulla* exhibited diversified associations including *Pseudomonas, Microbacterium, Pantoea, Phyllobacterium, Arthrobacter, Variovorax,* and *Phyllobacterium*. *Acacia* demonstrated also diverse associations with the genera *Pseudonocardia, Phyllobacterium, Devosia, Tardiphaga, Synechococcus, Paenibacillus, Bacillus, Ralstonia, Cohnella, Lysinibacillus,* and *Sphingobium*. The closest genera *Vachellia* is associated with *Bacillus, Pseudomonas, Microbacterium, Klebsiella, Stenotrophomonas*, and *Streptomyces*.

The analysis at the level of plant species, demonstrated that the genus *Pseudomonas* is prevalent in nodules of different leguminous species, like *Mimosa scabrella, Sulla coronaria, Sulla capitata*, and *Vachellia tortilis* subsp. *raddiana*. The genus *Enterobacter* was also found linked to various plant species, including *Crotalaria* sp., *Colophospermum mopane, Ormosia macrocalyx, Abrus precatorius*, *Mimosa pudica,* and *Acacia leiocalyx.* For its side, *Bacillus* was identified in nodules of *Erythrina brucei, Colophospermum mopane, Sphaerophysa salsula, Ormosia macrocalyx, Vavilovia formosa, Mimosa scabrella, Acacia disparrima, Acacia leiocalyx,* and *Vachellia tortilis* subsp*. raddiana;* while the closest genus *Paenibacillus* was found to be associated with nodules of *Indigofera* spp., *Erythrina brucei, Ormosia macrocalyx, Periandra mediterranea,* and *Acacia disparrima*. The genus *Serratia* was linked to *Erythrina brucei, Mimosa scabrella*, and *Mimosa pudica,* while*. Microbacterium* was found in nodules of *Colophospermum mopane, Sulla coronaria*, and *Vachellia tortilis* subsp. *raddiana. Phyllobacterium* was observed in *Sulla pallida, Sulla capitata, Acacia salicina, Acacia stenophylla,* and *Acacia leiocalyx*. Globally, these associations highlight the specificity and the diversity of nodule endophyte preferences across plant genera and species.

Furthermore, in the Supplementary material, a Sankey Diagram is employed to illustrate the principal plant subfamilies, alongside their associated plant genera and species. Another one delineates the primary plant genera and their associated plant species. These figures contribute Supplementary material visual insights, enhancing the comprehensive analysis presented earlier (Supplementary Figure S1).

## 4 Main properties of nodule endophytes

Endophytic bacteria are recognized for their role in promoting plant growth through mechanisms such as phytostimulation, biofertilization, and biocontrol (Batra et al., 2018). The hypothesis that nodule endophytes may exhibit similar beneficial actions is plausible. Specifically, endophytic bacteria residing in legume nodules demonstrate potential as biofertilizers. It has been demonstrated that some of them exhibit *in vitro* a range of advantageous activities for plants, including the synthesis of plant hormones, biological nitrogen fixation, and the solubilization of inorganic phosphate, as shown by Peix et al. (2015). Concurrently, others possess the catalytic activity of ACC deaminase or produce various compounds such as siderophores, as documented by (Ríos-Ruiz et al., 2019). Furthermore, certain nodule endophytic bacteria can enhance the well-being of their host plants by acquiring more restricted plant nutrients, as discussed by some authors (Leite et al., 2017; Freitas et al., 2019; Elhaissoufi et al., 2020).

Moreover, studies at the field level have focused on leveraging nodule endophytes to enhance the growth and adaptation of legumes to degraded estuarine soils. They have demonstrated that non-rhizobial nodule endophytes exhibit a significant potential to improve nodulation, modulate root exudation patterns, and foster legume growth (Dhole and Shelat, 2022; Debnath et al., 2023).

### 4.1 Biofertilization

#### 4.1.1 Nitrogen fixation by endophytes

Nitrogen fixation is a critical process for sustainable agriculture, and various bacterial strains have been identified for their ability to fix atmospheric nitrogen either as free-living organisms or as endophytes within legume nodules. Studies have documented the nitrogen-fixing capabilities of *Serratia* sp., *Herbaspirillum* sp., *Paenibacillus* sp., *Arthrobacter* sp., *Klebsiella* sp., *Pseudomonas* sp., *Bacillus* sp., *Bosea* sp., and *Enterobacter* sp. strains isolated from legume nodules (Valverde, 2003; Muresu et al., 2008; Selvakumar et al., 2008; Ibáñez et al., 2009; Knežević et al., 2021; Debnath et al., 2023).

Recent investigations suggest that NREs contribute substantially to nitrogen fixation, potentially accounting for up to 47% of the total nitrogen fixed by bacteria (Puri et al., 2018). This contribution spans both symbiotic and free-living states, underscoring the multifaceted role of NREs in nitrogen cycling within the biosphere.

#### 4.1.2 Phosphate solubilization for enhanced phosphorus availability

Phosphorus is a crucial element for plant growth that is often at low levels in the soils or present but not available for plants. At this respect, phosphate-solubilizing bacteria (PSB) play an essential role by transforming the insoluble molecules. It was reported that nodule endophytic bacteria can solubilize insoluble phosphates during initial colonization of roots, thereby improving phosphorus availability in the soil (Kai-yuan et al., 2023).

#### 4.1.3 Siderophore production as a mechanism for iron chelation

Certain strains of *Bacillus* within nodule endophytic bacteria contribute to plant growth by producing siderophores, which are low-molecular-weight metabolites that chelate iron, making it available to plants. This production has significant implications for competitively inhibiting the growth of phytopathogens (Maheshwari et al., 2019; Dhole and Shelat, 2022).

In addition to these traditional biofertilization mechanisms, recent studies have shed light on the role of nodular rhizosphere endophytes (NREs) in enhancing plant health and productivity. NREs, such as those isolated from soybean nodules, have been found to possess antimicrobial properties that extend beyond legume hosts, providing protection against bacterial and fungal pathogens in non-legume plants (Tokgöz et al., 2020).

### 4.2 Stress tolerance

Endophytes play an essentiel role in enhancing plant stress tolerance through various mechanisms, including the accumulation of osmolytes and antioxidant compounds, which aid in osmotic regulation and the stabilization of cellular components. Additionally, certain endophytic bacteria exhibit the ACC deaminase activity, an enzyme capable of degrading ACC and regulating ethylene production in plants, thereby promoting plant growth under stressful conditions (Vaishnav et al., 2019).

The significance of endophytic bacteria in stress tolerance has been underscored in several studies. For instance, Egamberdieva, (2017) demonstrated that *Achromobacter* spp., an endophyte associated with *Cicer arietinum* (chickpea), significantly improves plant growth promotion and confers potential salt/osmotic stress protection. Similarly, *Chryseobacterium* spp. has been shown to enhance osmotic stress protection in *Vigna unguiculata* (cowpea) (Leite et al., 2017). Moreover, certain endophytic bacteria such as *Enterobacter* sp. have exhibited remarkable abilities in abiotic stress adaptation, particularly in soybean and *Phaseolus lunatus* (Sharaf et al., 2019; Chibeba et al., 2020). Furthermore, Paenibacilli have been demonstrated to mitigate water-deficit stress in legumes by modulating stress-signaling hormones (Figueiredo et al., 2008).

Recent studies have shed light on the stress tolerance-inducing capabilities of non-rhizobial endophytic bacteria (NRBEs), particularly halotolerant strains. Jha et al. (2012) identified several halotolerant NRBEs, including *Brachybacterium* nov., *Zhihengliuella* sp., *Brevibacterium casei, Halomonas* sp., *Vibrio* sp., and *Pseudomonas* spp., which have shown potential in stress mitigation. Additionally, Mukhtar et al. (2020) highlighted the effectiveness of halotolerant NRBEs from cowpea nodules, such as *Pseudomonas fluorescens, Bacillus endophyticus, Bacillus pumilus*, and *Paenibacillus polymyxa*, in stimulating plant growth and development.

Furthermore, Ruiz-Díez et al. (2009) proposed that NRBEs, exemplified by *Phylobacterium myrsinacearum*, possessing plant growth-promoting abilities, can withstand acidic pH, thus potentially aiding in stress alleviation in acidic soil conditions. However, despite these advancements, there remains a notable gap in understanding the presence and potential contributions of bacterial endophytes in the small nodules of peanuts, particularly in facilitating plant growth promotion and tolerating biotic and abiotic stresses (Hossain et al., 2023).

### 4.3 Biocontrol

Pathogen diseases often develop resistance to chemical solutions, rendering them ineffective. Microorganisms, particularly those with plant growth-promoting characteristics, offer a promising approach for mitigating pathogen pressure (Horrigan et al., 2002; Kandel et al., 2017). In a study by Bahroun et al. (2018), three strains, namely *Rahnella aquatilis* B16C, *Pseudomonas yamanorum* B12, and *Pseudomonas fluorescens* B8P, identified as nodule endophytic bacteria, were assessed for their *in vivo* biocontrol potential against *F. solani* root rot in three Faba bean cultivars under greenhouse conditions. These strains significantly reduced pathogen symptom severity, with *R. aquatilis* B16C demonstrating the most effective protection across all tested Faba bean cultivars. Consequently, *R. aquatilis* B16C is recommended as a biocontrol agent for field application.

Mechanisms involved in the biocontrol of fungal diseases often implicate the production of lytic enzymes, with chitinases being particularly crucial. Sánchez-Cruz et al. (2019) reported that only the two *Serratia* sp. isolates (recognized as endophytic bacteria) exhibited chitinase production in their study, underscoring the importance of chitinase production for achieving biological control of phytopathogenic fungi (Berg and Hallmann, 2006; Wang et al., 2013).

In the study of Zhao et al. (2018), six endophytic antagonists belonging to five genera, including *Enterobacter, Acinetobacter, Pseudomonas, Ochrobactrum*, and *Bacillus*, were identified. Among them, *Acinetobacter calcoaceticus* DD161 displayed the strongest inhibitory activity (71.14%) against *Phytophtora. sojae* 01, inducing morphological abnormalities in fungal mycelia. Regression analysis indicated a significant positive correlation between siderophore production and the inhibition ratio against *P. sojae* 01.

In a recent study, Hnini et al. (2023) reported that four nodule bacterial endophytes, namely LMR701 (*Bacillus halotolerans*), LMR703 (*Pseudomonas koreensis*), LMR712 (*Bacillus thuringiensis*), and LMR713 (*Streptomyces tendae*), exhibited a antagonistic activity against *Fusarium oxysporum* and *Botrytis cinerea*. Similar results were observed with *Paenibacillus* strains (recognized as nodule endophytic bacteria), displaying antagonistic activities against five phytopathogenic fungi and synthesizing hydrolytic enzymes, siderophores, and the lipopeptide fusaricidin (Ali et al., 2022). When applied to plants, *B. subtilis* strain NUU4, a non–rhizobial endophytic bacterium, reduced diseased plants by 8%, highlighting the potential of such strains in significantly reducing plant diseases (Egamberdieva, 2017). Notably, nodule endophytic bacteria strains exhibiting significant lytic enzyme activities may play a crucial role in protecting plants against various pathogenic fungi, as indicated by studies on *Botrytis cinerea*, *Fusarium oxysporum*, *Pythium ultimum*, *Phytophthora* sp., and *Rhizoctonia solani* (Yasmin et al., 2016; Chen et al., 2020). The specific activities of these endophytic bacteria within nodules, such as induced systemic resistance (ISR), phytohormone production, biological nitrogen fixation (BNF), and stress tolerance, warrant further investigation.

It is uncertain whether the nodule endophytic can display in nodules all the biocontrol and bioprotection mechanisms expressed *in vitro.* However, endophytes in general (from other plant organs) are known to play diverse roles in supporting plant health by engaging in multifaceted interactions. Upon colonizing plant tissues, they participate in the competitive exclusion of pathogens by strategically occupying preferred sites of invasion, leading to the effective utilization of nutrients that curtails pathogen invasion (Xia et al., 2022). Another essential function involves antibiotic production, with certain strains, such as *Bacillus*, synthesizing compounds like circulin, colistin, and polymyxin. These compounds exhibit inhibitory effects on both Gram-positive and Gram-negative bacteria, as well as numerous pathogenic fungi. Additionally, several bacteria, including endophytes, can produce bacteriocins-specific proteins inhibiting the activity of strains within the same bacterial species or related species (Vasileva et al., 2019).

### 4.4 Phytostimulation

Plant hormones, crucial regulators of growth and development, encompass auxin, gibberellin, ethylene, abscisic acid, and cytokinin. Among them, indole-3-acetic acid (IAA) emerges as a crucial phytohormone produced by bacteria, significantly influencing root development and nutrient absorption. Noteworthy contributors to IAA production are endophytic bacteria across various genera, demonstrating positive impacts on plant growth and development by modulating biological processes such as fruit development, cell division, and aging (Batra et al., 2018). The IAA-producing capabilities of several endophytic genera, including those isolated from legume nodules (e.g., *Enterobacter, Pseudomonas, Klebsiella, Burkholderia, Pantoea, Bacillus,* and *Rhodococcus*), have been documented (Eid et al., 2021).

The question of the potential role of endophytes producing IAA in the establishment and functioning of symbiotic nodules is particularly intriguing. IAA is recognized for its essential role in symbiosis establishment and nodular tissue formation. In soybean, co-inoculation of endophytic bacteria with *Bradyrhizobium japonicum* MN110 enhances symbiotic nitrogen fixation and promotes plant growth, exemplifying the positive impact of endophytic bacteria on nodule function (Subramanian et al., 2015). Bacterial IAA, known for its multifaceted effects, extends its influence beyond simple growth modulation. It plays a critical role in enhancing nodule formation, boosting rhizobia competitiveness for nodulation, extending the functional lifespan of nodules by suppressing senescence, and stimulating the expression of genes associated with legume-rhizobia symbiosis (Alemneh et al., 2020). By promoting these mechanisms, bacterial IAA contributes significantly to the optimization of the legume-rhizobia symbiosis. Its role in facilitating nitrogen fixation in terrestrial ecosystems underscores its importance.

The hormone IAA is also responsible for the elongated root system and number of infection locations prior to nodulation. IAA is also involved in several stages of the symbiotic associations, and can also operate as a signal molecule in plant–bacteria associations and bacteria–bacteria communication (Etesami, 2022). Studies have shown that endophytic bacteria can improve nodule function and plant nitrogen in legumes, such as soybean, through synergistic interactions with rhizobia (Subramanian et al., 2015). In this study, authors found that co-inoculation of *Bradyrhizobium japonicum* MN110 along with endophytic bacteria *Bacillus megaterium* LNL6 and *Methylobacterium oryzae* CBMB20 exhibited an increase in nodule number and nodule activity, which was measured in terms of nodule leghemoglobin content, nodulated root ARA (acetylene reduction assay), and total plant nitrogen content compared to solitary inoculation of *B. japonicum* MN110. The high levels of IAA produced by *B. megaterium* LNL6 can be considered to have aided in the development of mature nodules, which thereby improved the nodular nitrogen fixation (Subramanian et al., 2015). The exploration of whether endophytes producing IAA actively contribute to nodule formation or modulate nodule function presents an avenue for valuable insights into the intricate interactions within legume-nodule associations. Moreover, the implications of hormones, including IAA, in plant stress tolerance raise pertinent questions regarding the potential role of endophytic bacteria within nodules in enhancing stress tolerance in leguminous plants. Investigating these potential roles could reveal novel mechanisms contributing to the resilience of legumes in challenging environments and deepen our understanding of the multifaceted functions of endophytic bacteria in the context of plant-microbe interactions.

### 4.5 Phytoremediation by endophytes

Plant-associated microbiomes play an essential role in phytoremediation, enabling plants to flourish in contaminated soils, alleviating stress induced by elevated levels of heavy metals and metalloids, and enhancing both phytoextraction and phytostabilization (Fagorzi et al., 2018). In addition to their biofertilizer potential cited before, legumes’ nodule endophytic bacteria were investigated for their role in phytoremediation of heavy metal-contaminated soils. In legumes, nitrogen-fixing bacteria and endophytes within nodules have demonstrated efficacy in reducing metal translocation to aerial plant parts, increasing plant nitrogen content, and promoting growth in metal-contaminated soils (Flores-Duarte et al., 2022b).

Under conditions of elevated metal levels in the soil causing stress to *Medicago sativa* (alfalfa), Adeleke et al. (2021) reported that inoculants based on non-rhizobial nodule endophytes could serve as useful and efficient tools to enhance legume adaptation and growth in metal-contaminated and nutrient-poor soils. Endophytes may offer advantages as plant growth-promoting bacteria (PGPB) compared to rhizospheric bacteria, owing to their closer association with the plant and reduced competition with soil bacteria.

Residual organic compounds accumulation in soils is another situation where the symbiotic relationship between plants and endophytic bacteria holds promise. Endophytic bacteria, isolated from environments rich in xenobiotics, particularly genera such as *Pseudomonas, Bacillus, Burkholderia, Stenotrophomonas, Micrococcus, Pantoea,* and *Microbacterium*, demonstrate versatile metabolic pathways enabling the utilization of organic pollutants as their sole carbon source. This capacity facilitates the mineralization or transformation of contaminants into non-toxic derivatives (Karaś et al., 2021).

## 5 Use of nodule endophytes as legume inoculants

Endophytic bacteria, with their diverse beneficial effects on plants, operate through mechanisms akin to plant growth-promoting rhizobacteria (Hallmann et al., 1997). Studies underscore the potential for increased yields across various plant species through endophyte inoculation (Pedraza et al., 2009). While historically focused on legumes, recent research suggests a broader applicability of nodule endophytes, prompting a reevaluation of their utilization criteria.

In the context of legumes, the combined application of rhizobial and non-rhizobial inocula has shown substantial yield increases, particularly when endophytes are compatible and growth inhibition is absent (Batra et al., 2018). Notably, the positive impact of early nodulation is exemplified in recent investigations on *Medicago sativa*, wherein co-inoculation with a consortium of endophytic strains led to superior nodulation and enhanced nitrogen content (Flores-Duarte et al., 2022b). Such findings highlight the potential synergistic benefits of co-inoculation with different endophytic strains in legume systems. However, endophytes’ influence on nodulation and yield varies across different symbioses. For example, while a *Bacillus* strain benefited the *R. tropici* - *Phaseolus vulgaris* symbiosis, it exhibited adverse effects in the *B. japonicum* - *Glycine max* symbiosis (Menéndez and Paço, 2020), Similarly, co-inoculation of *Vigna unguiculata* L. with *Bradyrhizobium* spp. and various non-rhizobial bacteria enhanced nitrogen levels and other parameters (Santos et al., 2018).

Beyond legumes, endophytic inoculation has shown remarkable benefits in crops like chickpeas, where enhanced antioxidant gene expression and growth were observed (El-Esawi et al., 2019). The application of *Bacillus subtilis* BERA 71 mitigated oxidative damage induced by salinity, further highlighting the potential of endophytes in stress tolerance (Abd_Allah Abd-Allah et al., 2018). Endophytes also influence osmotic processes, root morphology, and nitrogen metabolism in legumes (Dudeja et al., 2012). Furthermore, non-rhizobial endophytes (NREs) have positively influenced lentil growth, modulating root exudates and rhizospheric community structure (Debnath et al., 2023). Similarly, certain endophytes associated with peanut roots enhanced biomass production, demonstrating the broader impact of endophytes beyond nodulation (Hossain et al., 2023).

Studies in diverse agricultural systems highlight the positive effects of endophytic inoculation on plant growth and nutrient uptake (Minaxi et al., 2012; Lavakush et al., 2014; Ramesh et al., 2014; Freitas et al., 2019; Elhaissoufi et al., 2020). Notably, a nodule endophyte strain significantly enhanced root and nodule characteristics in common bean, influencing rhizosphere bacterial communities (Chihaoui et al., 2012, 2015), while historically considered primarily for legume inoculation, the utilization of nodule endophytes transcends plant families, offering promising avenues for enhancing biofertilizers’ efficacy in sustainable agriculture under various conditions. This evolving perspective underscores the need for a unifying criterion to maximize the potential benefits of nodule endophytes across diverse agricultural systems.

The interactions between endophytic bacteria and legumes elucidates several key highlights, including the enhancement of nitrogen fixation, improvement of plant growth and development, and the mitigation of stress in leguminous plants. Notably, various endophytic bacteria have demonstrated significant beneficial effects, such as increasing nitrogen levels, promoting root growth, and enhancing nutrient absorption in contaminated soils. These findings not only emphasize the versatility of endophytic bacteria across different legume species but also underscore their potential in addressing contemporary agricultural challenges, particularly in the realm of sustainable soil management and phytoremediation. By showcasing the multifaceted contributions of endophytic bacteria to legume health and productivity, the section provides compelling evidence of their importance in agricultural ecosystems and underscores the need for further research to unlock their full potential.

## 6 Insights from recent studies on new nodular endophytic bacteria

**Figure 9:**
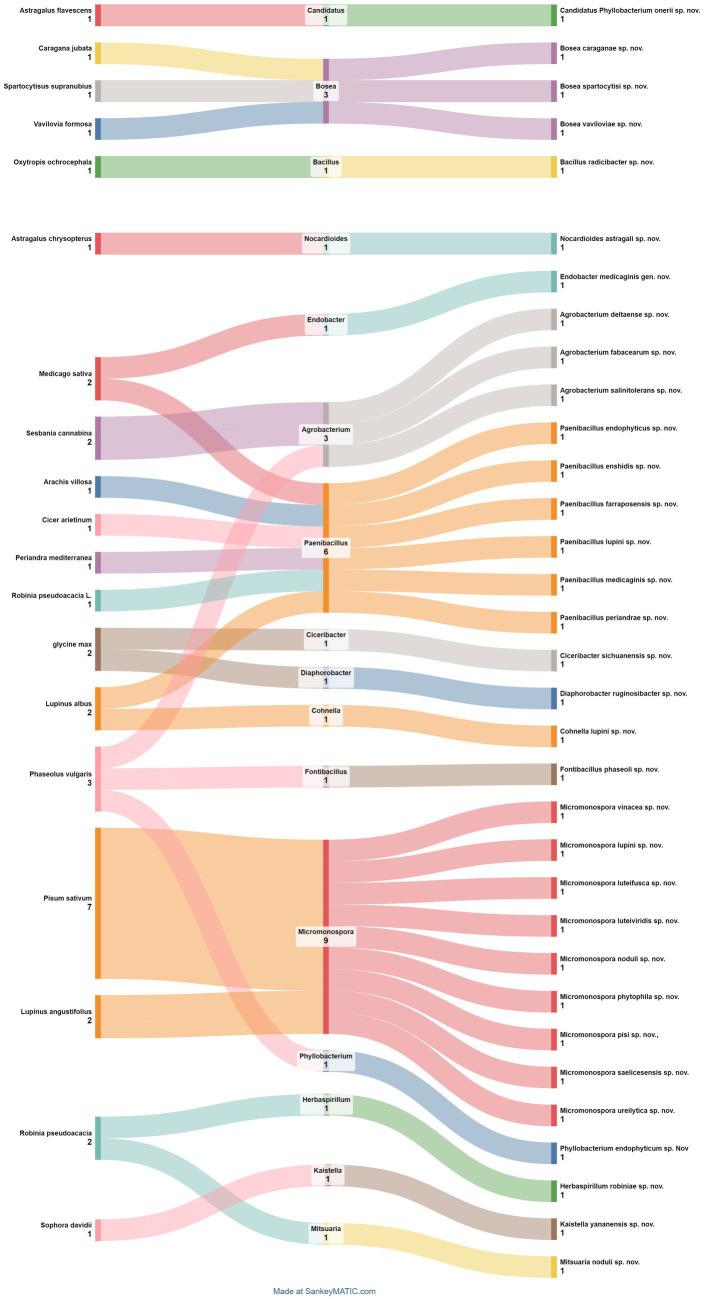
Sankey diagram representing the main plant species in this study along with the associated nodule endophyte genera and species.

The intricate world of nodular endophytic bacteria holds immense promise for researchers seeking to unravel the complexities of plant-microbe interactions. In this section, we present a meticulously curated compilation data (Table 2; Figure 9) summarizing recent studies on new species of nodular endophytic bacteria. This compilation serves as a valuable resource, offering insights into the diversity, distribution, and biotechnological potential of these intriguing microorganisms. Through diligent curation and analysis, we aim to provide a comprehensive overview while acknowledging the inherent limitations and nuances of our approach.

**Table 2:** Characteristics of endophytic *Bacteria* (sp. nov.) in legume nodules.

Species	Legumes	media for isolement	Origin	References
*Phyllobacterium endophyticum* sp. nov	*Phaseolus vulgaris*	*yeast extract-mannitol agar*	Salamanca (Spain).	Flores-Félix et al. (2013)
*Micromonospora phytophila* sp. nov. and *Micromonospora luteiviridis* sp. nov.	*Pisum sativum*	*yeast extract-mannitol agar*	Salamanca (Spain).	Carro et al. (2018)
*Kaistella yananensis* sp. nov.	*Sophora davidii*	*peptone yeast glucose (PYG) medium*	Shaanxi Province, China	Ai et al. (2024)
*Micromonospora pisi* sp. nov.	*Pisum sativum*	*yeast extract-mannitol agar*	Salamanca, Spain	Garcia et al. (2010)
*Paenibacillus endophyticus* sp. nov.	*Cicer arietinum*	*yeast extract-mannitol agar*	Salamanca, Spain	Carro et al. (2013)
*Paenibacillus lupini* sp. nov.	*Lupinus albus*	*yeast extract-mannitol agar*	Leon (Spain)	Carro et al. (2014)
*Micromonospora luteifusca* sp. nov.	*Pisum sativum*	*humic acid agar*	Cañizal, Zamora in Spain	Carro et al. (2016a)
*Micromonospora ureilytica* sp. *nov., Micromonospora noduli* sp. nov. and *Micromonospora vinacea* sp. nov.	*Pisum sativum*	*yeast extract-mannitol agar*	Cañizal (Spain)	Carro et al. (2016b)
*Agrobacterium fabacearum* sp. nov.	*Phaseolus vulgaris*	*yeast extract-mannitol agar*	Pallatanga/Chimborazo/Ecuador	Delamuta et al. (2020)
*Candidatus Phyllobacterium onerii* sp. nov.	*Astragalus flavescens*	*yeast extract-mannitol agar*	Izmir, Türkiye	Eren Eroğlu et al. (2024)
*Herbaspirillum robiniae* sp. *nov.*	*Robinia pseudoacacia*	*yeast extract-mannitol agar*	Shaanxi Province, China	Fan et al. (2018a)
*Mitsuaria noduli* sp. nov.	*Robinia pseudoacacia*	*yeast extract-mannitol agar*	Shaanxi Province, China	Fan et al. (2018b)
*Cohnella lupini* sp. nov.	*Lupinus albus*	*yeast extract-mannitol agar*	Leon (Spain)	Flores-Félix et al. (2014a)
*Fontibacillus phaseoli* sp. nov.	*Phaseolus vulgaris*	*yeast extract-mannitol agar*	Spain	Flores-Félix et al. (2014b)
*Paenibacillus medicaginis* sp. nov.	*Medicago sativa* L.	*yeast extract-mannitol agar*	Taiwan	Lai et al. (2015)
*Paenibacillus periandrae* sp. nov.	*Periandra mediterranea*	*yeast extract-mannitol agar*	Brazil	Menéndez et al. (2016)
*Bosea spartocytisi* sp. nov.	*Spartocytisus supranubius*	*yeast extract-mannitol agar*	Teide National Park (Canary Islands)	Pulido-Suárez et al. (2022)
*Endobacter medicaginis* gen. Nov.	*Medicago sativa*	*yeast extract-mannitol agar*	Zamora, Spain	Ramírez-Bahena et al. (2013)
*Bosea vaviloviae* sp. nov.	*Vavilovia formosa*	*(YMA)* (Vincent, 1970) *supplemented with 0.5% succinate*	North Ossetia	Safronova et al. (2015)
*Bosea caraganae* sp. nov.	Caragana jubata	*(YMA)* (Vincent, 1970) *supplemented with 0.5% succinate*	Mongolia	Sazanova et al. (2019)
*Micromonospora lupini* sp. nov. and *Micromonospora saelicesensis* sp. nov.	*Lupinus angustifolius*	*Yeast extract-mannitol agar*	Salamanca, Spain	Trujillo et al. (2007)
*Diaphorobacter ruginosibacter* sp. nov.	*Glycine max*	*yeast extract-mannitol agar*	Shaanxi, China	Wei et al. (2015a)
*Bacillus radicibacter* sp. nov.	*Oxytropis ochrocephala*	*yeast extract-mannitol agar*	Qilian mountain, China.	Wei et al. (2015b)
*Nocardioides astragali* sp. nov.	*Astragalus chrysopterus*	*yeast extract-mannitol agar*	northwestern China	Xu et al. (2018)
*Agrobacterium deltaense* sp. nov.	*Sesbania cannabina*	*yeast extract-mannitol agar*	Shandong Province of China	Yan et al. (2017a)
*Agrobacterium salinitolerans* sp. nov.	*Sesbania cannabina*	*yeast extract-mannitol agar*	Shandong Province of China	Yan et al. (2017b)
*Paenibacillus enshidis* sp. nov.	*Robinia pseudoacacia* L.	*yeast extract-mannitol agar*	Hubei, PR China	Yin et al. (2015)
*Paenibacillus farraposensis* sp. nov.	*Arachis villosa*	*yeast extract-mannitol agar*	Río Negro, Uruguay.	Roldán et al. (2022)
*Ciceribacter sichuanensis* sp. nov.	*Glycine max*	*yeast extract-mannitol agar*	Sichuan, China	Zhang et al. (2024)

The compilation data serves as a succinct summary of recent studies documenting new species of nodular endophytic bacteria. We recognize the significance of this compilation and have diligently ensured the clarity and comprehensiveness of the presented data. Key details such as the host legumes, isolation media, and geographical origins of the newly identified species are meticulously documented, facilitating a thorough understanding of nodular diversity.

We firmly believe that this compilation table serves as an invaluable resource for researchers interested in delving into the diversity and biotechnological potential of nodular endophytes. By consolidating findings from diverse studies, the table offers valuable insights into the distribution of nodular endophytic bacteria across various legume hosts and geographical locations. Furthermore, the inclusion of references enables easy access to the primary sources, thereby augmenting the utility of the table for readers. While the compilation table provides a comprehensive overview of newly described nodular endophytic bacteria, we acknowledge potential limitations inherent in our approach. These limitations, including geographic biases and potential underrepresentation of certain legume hosts or regions in the literature, have been duly addressed through discussions regarding the scope and focus of our literature search. We have also highlighted the potential implications of these limitations for the interpretation of our findings.

Additionally, we have initiated a concise discussion on the biotechnological potential of the newly described species, elucidating their unique characteristics and potential applications. By juxtaposing these species with previously known nodular endophytes, we aim to discern any distinctive features or evolutionary trends contributing to their biotechnological relevance.

Regarding the geographical distribution of research efforts, Spain emerges as a prominent contributor, with 11 studies and 15 new species of endophytic nodule bacteria, followed closely by China with 7 studies and 7 new species. *Micromonospora* leads among bacterial genera, with 9 new species (7 from *Pisum sativum* and 2 from *Lupinus angustifolius*), followed by *Paenibacillus* with 6 new species from different plant species. Subsequently, there are 3 new species each of *Agrobacterium* and *Bosea*.

It is evident that while there are variations in the media used for isolating certain species, the majority of new species (25 out of 31) were isolated using yeast extract-mannitol agar (YMA) (Vincent, 1970). This underscores the widespread utilization and efficacy of YMA for isolating a diverse array of bacterial species, including those associated with nodules and endophytes. However, it is noteworthy that specific media formulations were employed for isolating certain species, such as YMA supplemented with 0.5% succinate for *Bosea* sp., humic acid agar for *Micromonospora luteifusca* sp. nov. These variations likely arise from the unique requirements or characteristics of these particular bacteria.

In conclusion, while yeast extract-mannitol agar remains a popular choice for bacterial isolation, researchers may opt for specialized media formulations when targeting specific bacterial groups or when seeking to optimize isolation conditions for particular species.

## 7 Exploring microbial diversity in root nodules: integrating cultivation-based and metagenomic approaches

It’s essential to recognize the constraints of cultivation-based techniques in capturing the entirety of microbial communities. While culturomic methods provide valuable insights into cultivable microorganisms, they inherently overlook the vast majority of unculturable diversity present in environmental samples. To bridge this gap, molecular approaches, particularly metagenomics, offer a more comprehensive understanding of microbial diversity by analyzing total DNA isolated from environmental samples.

The findings from various metagenomic studies exemplify the utility of metagenomic approaches in elucidating the diversity of endophytic populations associated with root nodules. For instance, Hakim et al. (2020) identified a diverse array of bacterial genera, including non-rhizobia such as *Acinetobacter, Enterobacter,* and *Pseudomonas*, in mung bean nodules, emphasizing the importance of considering non-rhizobial taxa and their potential contributions to plant growth promotion and symbiotic interactions. Similarly, Iyer and Rajkumar (2017) utilized metagenomic approaches to characterize bacterial communities in the rhizosphere, root endosphere, and root nodules of *Vigna radiata.* Their findings highlighted the predominance of *Proteobacteria* in rhizospheric and nodular communities, with *Actinobacteria* being predominant in the root endosphere, demonstrating the distinct microbial compositions across different plant-associated habitats.

Moreover, the study conducted by Jesus et al. (2023) utilized a combination of histological analysis and Next-Generation Sequencing to assess nodular structure and biodiversity in *Acacia longifolia*. Their findings revealed shifts in microbial community composition following fire events, with *Bradyrhizobium* and cyanobacteria being predominant in both burnt and unburnt sites, underscoring the dynamic nature of microbial communities within nodules and their potential responses to environmental perturbations.

Additionally, Sharaf et al. (2019) provided insights into the nodule bacteriome of soybean cultivars using metagenomic approaches. Their results highlighted the prevalence of non-rhizobial taxa such as *Pseudomonas* and *Enterobacteria*, suggesting a significant contribution of these taxa to the nodule community, further emphasizing the importance of considering the broader microbial diversity within nodules and its implications for plant-microbe interactions.

In addition to the studies previously mentioned, it’s important to consider the source of nodular endophytes (NRE) and the limitations associated with metagenomic analysis of bacterial community diversity. Bender et al. (2022) discussed how genera like *Rhizobium, Pseudomonas,* and *Klebsiella* can be transferred vertically from seeds to the root system of legumes, suggesting potential vertical transmission mechanisms. However, in non-sterile soils, it’s challenging to confirm whether these bacteria are acquired through horizontal or vertical transmission. The entry of non-rhizobia into nodules may occur through the junction between the nodule and root or via infection threads induced by rhizobia (Zgadzaj et al., 2015). The true endophytic nature of NRE can only be determined through fluorescent labeling or isolation of viable cells from surface-sterilized nodules to prevent contamination from attached bacteria (Hakim et al., 2020).

Moreover, while metagenomic analysis offers advantages over culture-based techniques, it has limitations in terms of the quality and quantity of metagenomic DNA sampling. Cultivation-independent molecular strategies, such as generating 16S rRNA libraries and phylogenetic analysis, can augment our understanding of unculturable microbiota in the rhizosphere and their interactions with plants and other microorganisms (Iyer and Rajkumar, 2017). However, technological barriers hinder meticulous analysis despite the advancements in “omics” techniques. Therefore, addressing these limitations and integrating multiple approaches are crucial for advancing our understanding of microbial diversity and its implications for plant-microbe interactions and agricultural sustainability.

To sum up, while culturomic approaches offer valuable insights, integrating metagenomic studies provides a more comprehensive understanding of nodular diversity and its biotechnological potential. These studies collectively underscore the necessity of incorporating both cultivation-independent and cultivation-based techniques to gain a holistic view of microbial communities associated with root nodules.

## 8 Conclusion

In conclusion, this review provides comprehensive insights into the intricate relationships between various legume plant genera and their associated nodule endophytes. The study highlights the specificity and diversity of nodule endophyte preferences across various plant genera, underscoring the complexity of plant-endophyte interactions. The investigation reveals diverse prevalence patterns, emphasizing the significance of certain genera such as “*Phaseolus*” and the consistent presence of *Bacillus* and *Pseudomonas* as predominant nodule endophyte bacteria. Legume nodules, complex habitats for diverse bacterial communities, exhibit a prevalence of specific genera, notably *Pseudomonas* and *Bacillus*. Beyond rhizobia, non-rhizobial endophytes contribute to the ecological resilience of legumes. These findings have implications for agricultural practices, suggesting potential applications in targeted inoculation to enhance plant growth, nutrient uptake, plant resilience and overall health in different legume crops under different soil and climatic conditions.

## Author contributions

MH: Conceptualization, Data curation, Formal analysis, Investigation, Methodology, Software, Writing – original draft. JA: Conceptualization, Formal analysis, Project administration, Resources, Supervision, Validation, Writing – review & editing.

## Supplementary material

SUPPLEMENTARY FIGURE 1 Sankey diagram represents the main plant subfamilies in this study along with the associated plant genus and species.
