# Supplementary figures and images for: Prevalence, diversity and applications potential of nodules endophytic bacteria: a systematic review
Source: Front Microbiol. 2024 May 15;15:1386742. doi: 10.3389/fmicb.2024.1386742 (PMC11133547; doi:10.3389/fmicb.2024.1386742)

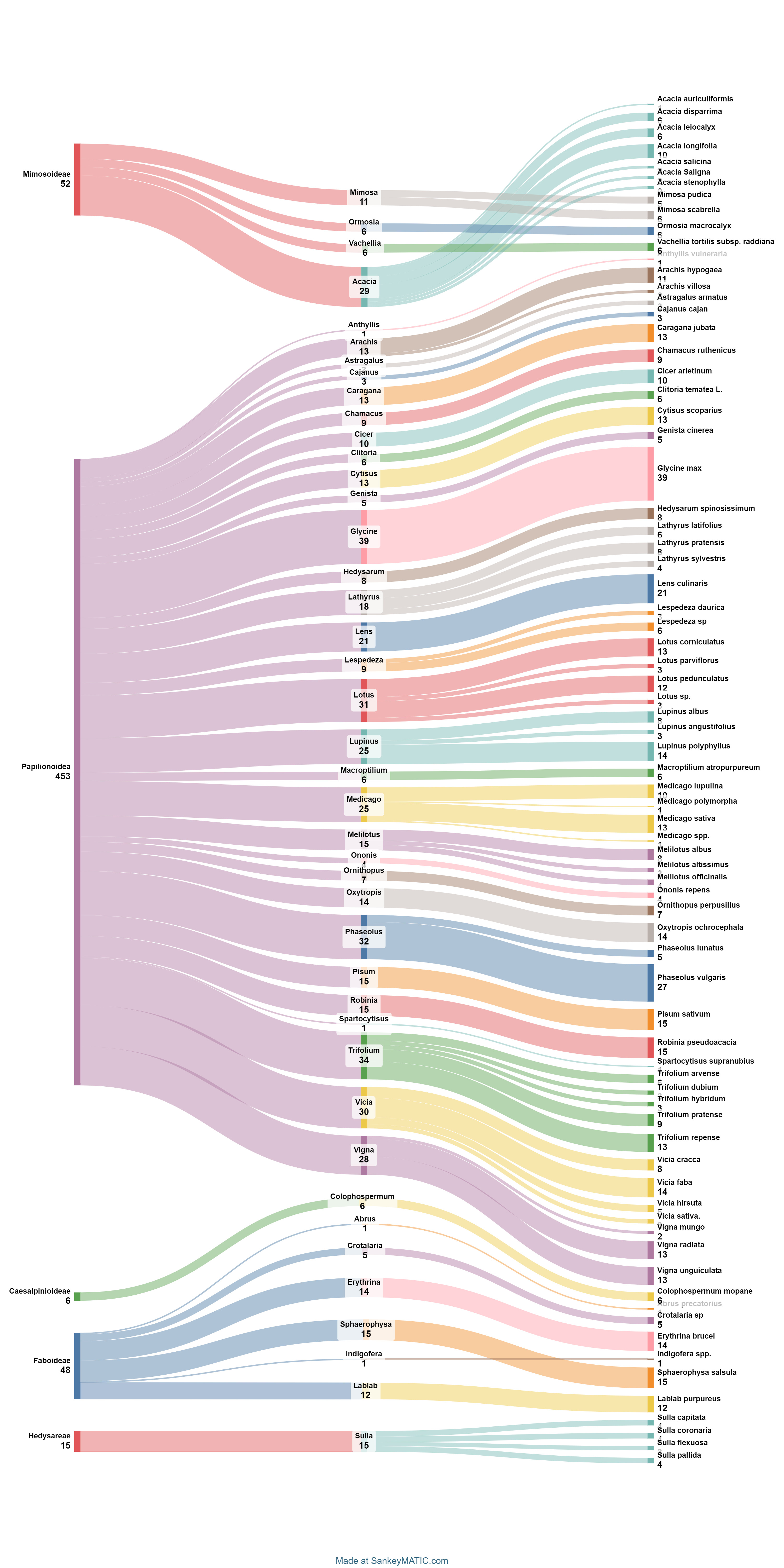

Supplement: SUPPLEMENTARY FIGURE 1 — Sankey diagram represents the main plant subfamilies in this study along with the associated plant genus and species. [file Image_1.jpeg]
